# Mental Health and Related Factors Among Undergraduate Students During SARS-CoV-2 Pandemic: A Cross-Sectional Study

**DOI:** 10.3389/fpsyt.2022.833263

**Published:** 2022-05-31

**Authors:** José Miguel Valdés, Francisco Javier Díaz, Pascale Marie Christiansen, Gabriel Arturo Lorca, Francisco Javier Solorza, Matías Alvear, Saray Ramírez, Daniel Nuñez, Ricardo Araya, Jorge Gaete

**Affiliations:** ^1^School of Medicine, Universidad de los Andes, Santiago, Chile; ^2^Faculty of Education, Research Center for Students Mental Health (ISME), Universidad de los Andes, Santiago, Chile; ^3^National Research and Development Agency (ANID), Millennium Science Initiative Program, Millennium Nucleus to Improve the Mental Health of Adolescents and Youths, Imhay, Santiago, Chile; ^4^Faculty of Psychology, Universidad de Talca, Talca, Chile; ^5^Department of Health Service and Population Research, King's College London, London, United Kingdom

**Keywords:** mental health, undergraduate, college, depression, anxiety, suicide risk, university, insomnia

## Abstract

**Background:**

Mental health problems among undergraduates are a significant public health concern. Most studies exploring mental health in this population during the pandemic have been conducted in high-income countries. Fewer studies come from Latin American countries. The aim of this study was to determine the prevalence of depression, anxiety, stress, insomnia, and suicide risk, and explore the association with several relevant variables in personal, family, university, and SARS-CoV-2 pandemic domains.

**Methods:**

A cross-sectional study was conducted in Chile in a medium-size private University. Outcome variables were explored with valid instruments: Depression, Anxiety, and Stress Scale (DASS-21), Insomnia Severity Index (ISI), and the Columbia-Suicide Severity Rating Scale (C-SSRS). Independent variables from personal (e.g., sex, age, sexual orientation, history of mental health problems, substance use), family (e.g., parental educational background, family history of mental health problems, family functioning), university (e.g., course year, financial support, psychological sense of university belonging, history of failing subjects) and SARS-CoV-2 domains (e.g., history of personal and family contagion, fear of contracting SARS-CoV-2, frequency of physical activity, keeping routines and social contact). Multivariable logistic regression models were conducted for each outcome, after univariable and domain-specific multivariable models. The significant variable at each step was selected if the *p*-value was ≤ 0.05.

**Results:**

A total of 5,037 students answered the survey—the global response rate of 63.5%. Most of the students were females (70.4%) and freshmen students (25.2%). The prevalence of mental health problems was high: depression (37.1%), anxiety (37.9%), and stress (54.6%). Insomnia was reported in 32.5% of students, and suicide risk in 20.4% of students. The associated variables at personal domain were history of mental health problems, substance use, and sexual orientation; at family domain, family functioning and family history of mental health problems; at university domain, violence victimization and sense of belonging; and in SARS-CoV-2 domain, having a daily routine and fear to contracting SARS-CoV-2 by students themselves or others.

**Conclusions:**

The prevalence of mental health problems is high among undergraduate students and some of the associated factors, such as victimization and a sense of belonging can be used in preventive interventions.

## Introduction

Mental health problems are considered a significant public health concern, especially among young people. Pre-pandemic studies have estimated the prevalence of mental disorders among youth from 8.3 to 12.4% (1) and concerning figures can also be seen among undergraduate students. For instance, in USA, The Healthy Minds Study (2020) found a 39% prevalence of depression and a 34% prevalence of anxiety disorder among college students (2). In Chile, the prevalence of mental health problems among undergraduate students seems to have increased in the last few years (3). For instance, in 2013, Antúnez et al., using the Depression, Anxiety, and Stress Scale (DASS-21), found that 30% of undergraduates had depressive symptoms and 21% had anxiety symptoms (4), while, in 2019, Barrera-Herrera et al., using the same instrument, found that both depression and anxiety reached a 46% prevalence (5).

Young people face a crucial developmental period with many normative transitions. Vocational issues, financial problems, academic workload, missing home, and loneliness are some of the common stressors found among undergraduate students (1, 6, 7). The changes found in this period may be aggravated by SARS-CoV-2-related stressors and social disruptions associated with the restrictions in daily activities imposed by the pandemic (8).

Several studies have explored the effect of SARS-CoV-2 pandemic and sanitary measures on mental health among undergraduates. In the USA, 48.1% of undergraduates reported depression, 38.5% anxiety, and 18.0% suicidal ideation. In the same study, 71.3% of the students indicated that their stress and anxiety symptoms increased during the pandemic (9). Additionally, a recent meta-analysis estimated a mean prevalence of 31% for anxiety and 34% for depression (8). Anxiety was slightly higher among males (36%) and females (30%), but depression was higher among females (56%) than among males (34%).

Mental health problems may disrupt performance in the University. For example, a study conducted in USA found that during the pandemic high levels of depression were associated with difficulties in focusing on academic work (10). Other findings demonstrated that mental health distress was correlated to a perceived poor academic performance caused by SARS-CoV-2 (11).

Several factors have been identified related to the presence of mental health problems, some of them related to the expansion of the disease and other related to personal or contextual factors. In the first case, a large survey conducted in China found an increased risk of mental health problems when undergraduates reported having relatives or friends being infected with SARS-CoV-2 (12). A study in Taiwan showed that self-reported susceptibility of contracting SARS-CoV-2 was associated with suicidal ideation (13). In the second case, a study conducted in Canada reported that females had higher rates of mental health problems (14). On the other hand, one study conducted in Indonesia, Taiwan, and Thailand showed found that a higher support received from family, classmates, and faculties decreased suicidal ideation (15). A study in USA found that a higher alcohol use was associated with increased depression and anxiety symptoms (16). A study in Spain reported that using tobacco, insomnia and low self-esteem were associated with depression, anxiety and stress (17). Another large survey conducted in France showed that low-quality housing and insolation were highly associated with mental health problems (18).

In Latin America, fewer studies have explored the effect of the pandemic on mental health among undergraduates. It is known that in this region of the world higher mental problem symptoms have been reported in the general population during the pandemic (19), and also before the pandemic for various reasons such as treatment gap (20). We have found three studies conducted in Brazil (21–23), two studies conducted in Mexico (24, 25), and one in Chile (26). The studies in Brazil found that undergraduate students exhibited higher scores for perceived stress and depressive signs and lower resilience scores when compared to administrative and faculty staff (21). Additionally, variables associated with symptoms of depression, anxiety and stress in Brazil were: being female, having a chronic disease, fewer positive relations with others, lower self-acceptance, self-blaming, and substance use (22, 23). The studies in Mexico found that anxiety and depressive symptoms were related to younger age, previously diagnosed psychiatric disorder, and using drugs (25); and the main reason undergraduate students requested professional help was the presence of anxiety symptoms (24). Finally, only one research explored mental health problems among first-year university students from a State University in Chile, and found that 77% of the students perceived that their mood was worse during the pandemic, and the main associated factors with both depressive and anxiety symptoms were being female, problems with family or friends, problems with online classes and attention deficit symptoms (26).

The general aim of this study was to determine the prevalence of mental health problems among undergraduate students and is associated factors among undergraduate students in Santiago, Chile. The specific aims were: First, to determine the prevalence of depression, anxiety, stress, insomnia, and suicide risk. Second, to explore the association of personal, family, and university variables with mental health outcomes. And third, to estimate the association between SARS-CoV-2 pandemic and sanitary measures on the mental health of students.

## Methods

### Participants

A cross-sectional study based on individual-level data was conducted using an online survey. The protocol of the study was approved by the Ethical Committee of Universidad de los Andes (CEC201984) and registered (ClinicalTrials.gov NCT04447690).

This study followed a convenience sampling strategy. The inclusion criteria were: (1) Registered undergraduate students from Universidad de los Andes, Santiago, Chile, and (2) 18 years old and older at the time of the study. All eligible students (*n* = 7,935), were invited to answer an online survey sent to their institutional email address using Qualtrics, ensuring anonymity.

### Procedure

We contacted all authorities of different Faculties and Academic Units, and a written and signed letter of authorization was required for participation. A massive campaign using social media networks and institutional emails was conducted between June 11th and September 4th 2020. Special efforts were directed to contact and ask for support from social media influencers who actively invited students to answer the survey. Academic councils and authorities were also asked to share information about the study and the survey link.

All relevant information about the study, such as objectives of the study, potential benefits and risks, as well as confidentially issues, the written consent and the link to answer the survey was send to the students using the institutional emails and the formal channels of communication between Faculties and Academic Units and students. As a result, participants were asked to confirm or refuse to answer the survey. Data entry was possible between August 5th and September 4th 2020. Regarding the pandemic context, it is important to mention that Chile was in the 5th month after the first case reported of SARS-CoV-2 and the Government of Chile implemented lockdown measures in all the country which were in place at the moment of the data collection. Therefore, universities were only implementing online classes. No vaccines were available at that time in Chile.

### Privacy and Anticipation of Potential Risks

Anonymity was ensured and answers were not traceable. In order to promote help-seeking actions among students who considered themselves to have mental health symptoms, at the end of the survey, self-help reflexive questions and phone numbers of several public institutions were displayed. There, they could ask for help and access assistance if needed. Additionally, high suicide risk was detected using the Columbia-Suicide Severity Rating Scale (Answering “yes” to questions #4 or #5). If high risk was detected, a warning sign appeared on the screen suggesting seeking help, alongside the university wellbeing support contact email address and the phone number of several public institutions where help can be obtained. Every week we informed the university wellbeing personnel on the number of students who potentially may contact them during the following week so they can be prepared to take care of them. Due to the fact that no names or identity information were obtained in the survey, this information was never informed to third parties. Finally, the study opened an Instagram account (http://www.instagram.com/salud_mental_med_uandes) where we shared self-help information and provided contact email addresses and contact phone numbers of wellbeing personnel for those students who may require assistance.

To prevent multiple answers, only institutional mailing systems were used, and a record of IP addresses blocked further attempts to answer multiple times. These IP addresses were encrypted in the server.

### Measures

The survey included widely used scales and, when not instruments were available, several questions were developed by the researchers.

#### Independent Variables

Variables from four domains were included in the questionnaire.

##### Personal Domain

- **Sociodemographic variables**. Sex, age, nationality (0 = Chilean; 1 = Other nationality), ethnicity (0 = Non-indigenous; 1 = Indigenous), and occupational status (0 = Studying only; 1 = Studying and part-time job; 2 = Studying and full-time job).
- **General health history**. History of chronic illness (0 = No; 1 = Yes), history of mental health disorders (Depression, Bipolar, Panic, Anxiety, Eating, Attention Deficit Disorder w/Hyperactivity-ADHD), history of mental health treatment (Psychotherapy, Pharmacotherapy), physical activity (0, ≤ 149 min/week; 1, ≥150 min/week. This is equivalent of performing psychical exercise 30 min/day five times a week).
- **Sexuality and sexual health**. Sexual orientation (Heterosexual, Homosexual, Bisexual, Unsure, Other), offspring (0 = No children; 1 = With children), number of sex partners in the last year (0 = None; 1 = 1 or 2; 2 = 3 or more).
- **Substance use:** We used two screening questionnaires: the CAGE questionnaire (27) for alcohol use, and an adapted version of the Alcohol, Smoking and Substance Involvement Screen Test (ASSIST) (28), which included all substances of abuse, but alcohol. We decided to use CAGE for alcohol use because it is a self-report 4-item scale used to assess harmful alcohol consumption (abuse or dependence) widely used in Chile and allowed us to reduce the number of items to the survey. Each item is answered 0 (*No*) or 1 (*Yes*). The total range score goes from 0 to 4. The cut-off point is ≥2 (27). It is able to detect alcohol abuse and dependence with a sensitivity of 43%-94% and a specificity of 70–97% (29). It has been validated in Spanish populations (30). The ASSIST is an interview with eight items exploring the amount and frequency of substance use in the last 3 months and the problems associated with its use. Originally, it explores ten drugs (Tobacco, alcohol, marijuana, cocaine, amphetamines, inhalants, sedatives, hallucinogens, opioids, and others). We included “Vaping” and the use of “nootropics” (cognitive enhancers) in the list of drugs because of the recent concern of increased use among undergraduate students. We adapted the ASSIST to be used as a self-report questionnaire. Additionally, alcohol questions were excluded from this instrument because this substance was assessed with the CAGE for the reasons mentioned above. For all substances, we reported the prevalence of use in the last week, last month, lifetime, and risk of substance use disorder. For the association analyses, we only used the monthly use (0 = No use in the last month; 1 = Monthly use) of the most frequent substances.

##### Family Domain

- **Family history**. Parental educational level (0 = Incomplete elementary school; 1 = Complete elementary school and incomplete high school; 2 = Complete high school and incomplete university; 3 = Complete university and incomplete postgraduate studies; 4 = Complete postgraduate studies), family history of mental health problems (Psychiatric disorders, Suicide, Alcohol abuse or dependence, and Drug abuse or dependence).
- **Family functioning**. We used the “Family functionality APGAR score”. This tool is a self-report questionnaire of 5 items, with responses on a Likert Scale from 0 (*Almost never*) to 2 (*Almost every time*) that measures family support in the domains of adaptation, partnership, growth, affection, and conflict resolution. It is interpreted as follows: Highly functional family: 7–10; Moderate dysfunction: 4–6; Severe dysfunction: 0–3 (31). Cronbach's alpha in the Spanish population is 0.84) (32). Cronbach's alpha in our sample was 0.87. The reference category for association analyses was Highly functional family.

##### University Domain

- **University history**. Academic year (1 = First year; 2 = Second year; 3 = Third year; 4 = Fourth year; 5 = Fifth or more), source of financing (Funding from parents, Credit/loan, Scholarship, Self-funded, Other means), mean commuting time (1 = Higher than average (≥1SD); 2 = Average (of the total sample) (reference group); and 3 = Lower than average ( ≤ 1SD), history of failing subjects (0 = No failed subjects; 1 = Failed subjects).
- **Violence victimization**. The research team developed several questions aiming to gather information about violence victimization: physical, psychological, exclusion, teasing, and ridiculization.
- **Psychological sense of university belonging** was measured using the Sense of Social and Academic Fit tool (SSAF), previously validated in English (33). The tool is a self-report questionnaire of 17 items, with responses on a Likert Scale from 1 (*Strongly disagree*) to 7 (*Strongly agree*) that measure academic and social sense of belonging. Cronbach's alpha in this sample was 0.90; the higher the score, the higher sense of belonging. We categorized this variable according to three groups: 1 = Higher than average (≥1SD); 2 = Average (of the total sample) (reference group); and 3 = Lower than average ( ≤ 1SD).

##### SARS-CoV-2 Experiences Domain

- **SARS-CoV-2 experiences**. History of personal (0 = No; 1 = Yes) and family (0 = No; 1 = Yes) contagion of SARS-CoV-2; fear to contracting SARS-CoV-2 by the students (1 = Not at all; 2 = Slightly; 3 = Somewhat; 4 = Moderately; 5 = Extremely) and fear of others contracting SARS-CoV-2 (1 = Not at all to 5 = Extremely); living condition during pandemic lockdowns (0 = Living with family, friends or roommates; 1 = Living independently); frequency of social contact during lockdowns (1 = Never; 2 = 1 or 2 days/week; 3 = 3 or 4 days/week; 4 = 5 or 6 days/week; 5 = Everyday); frequency of physical exercising during lockdowns (1 = Never to 5 = Everyday); frequency of recreational activities during lockdowns (1 = Never to 5 = Everyday); keeping a routine during lockdowns (1 = Never to 5 = Everyday); and frequency of meditation or praying during lockdowns (1 = Never to 5 = Everyday).

#### Dependent Variables

- **Depression, anxiety and stress**. We used the Depression, Anxiety, and Stress Scale (DASS-21) (34, 35). This instrument has 21 items divided into three subscales, and it has been validated in the Chilean college population (36). The cut-off score for the depression subscale is ≥6 (sensitivity 88.46% and specificity 86.77%), for the anxiety subscale is ≥5 (sensitivity 87.50% and specificity 83.38%), and for the stress subscale is ≥6 (sensitivity 81.48% and specificity 71.36%). We used these cut-off scores to create binary outcome variables. The reported Cronbach's alpha for each subscale is 0.88, 0.71, and 0.80, respectively (37). Cronbach's alpha in our sample for depression subscale was 0.89, for anxiety subscale was 0.83, and for stress subscale was 0.88.
- **Insomnia**. We used the Insomnia Severity Index (ISI) (38). It is a 7-item scale with answers ranging from 0 to 4. A higher score means more severe symptoms of insomnia. The cut-off score for clinical insomnia is ≥15 (38). This cut-off score was used to create a binary outcome variable. The English validation reported a Cronbach's alpha of 0.90 (39), and the Spanish validation reported a Cronbach alpha of 0.82 (40). Cronbach's alpha in our sample was 0.84.
- **Suicide risk**. We used the Columbia-Suicide Severity Rating Scale (C-SSRS) (41). This scale has seven items exploring the presence of suicidal ideation and plans and suicide attempts in different periods of time. Each item is responded Yes or No. It has been validated in English-speaking (Cronbach's alpha between 0.73 and 0.93) (41), and Spanish speaking populations (α = 0.53) (42). For the analysis of this study, we only considered the first five items that measured suicide risk in the last month. Cronbach's alpha in our sample was 0.74. We created a binary variable (0 = No risk; 1 = Suicide risk) as outcome.

### Statistical Analyses

A descriptive analysis was performed with measures of variance by calculating 95% confidence intervals and standard deviation accordantly. Measures of central tendency were calculated with the mean, and relative frequencies and percentages were presented (see Table 1).

**Table 1:** Personal variables.

**Personal variables**	***n***	**% or mean**	**(95% CI) or (SD)**
**Sex**			
Male	1,491	29.6	(28.4–30.9)
Female	3,546	70.4	(69.1–71.6)
**Age distribution by academic year**			
1st	1,268	19.6	(2.9)
2nd	961	20.6	(2.7)
3rd	992	21.5	(1.8)
4th	936	22.4	(1.6)
5th or higher	880	24.0	(1.9)
**Nationality**			
Chilean	4,860	96.5	(95.9–97.0)
Other	177	3.5	(3.0–4.1)
**Indigenous ethnicity**			
Aimara	6	0.12	(0.1–0.3)
Atacameño	2	0.04	(0.0–0.2)
Collas	1	0.02	(0.0–0.1)
Diaguita	5	0.10	(0.0–0.2)
Mapuche	73	1.51	(1.2–1.9)
Quechua	1	0.02	(0.0–0.1)
Easter islander	1	0.02	(0.0–0.1)
Yamana	2	0.04	(0.0–0.2)
Total	91	1.9	(1.5–2.3)
**Occupational status**			
Studying only	4,224	87.5	(86.5–88.4)
Studying and part-time job	541	11.2	(10.3–12.1)
Studying and full-time job	62	1.3	(1.0–1.6)
**History of chronic illness**			
Yes	858	19.4	(18.3–20.6)
No	3,567	80.6	(79.4–81.7)
**History of mental health disorders**			
Depression	1,237	27.9	(26.6–29.3)
Bipolar disorder	135	3.1	(2.6–3.6)
Panic disorders	993	22.4	(21.2–23.7)
Anxiety disorders	1,568	35.4	(34.0–36.8)
Eating disorders	448	10.1	(9.3–11.0)
ADHD	1,447	32.7	(31.3–34.0)
**History of mental health treatment**			
Psychotherapy	2,013	45.4	(44.0–46.9)
Pharmacologic	1,580	35.7	(34.3–37.1)
**Physical activity during the week**			
≥150 min	1,932	43.7	(42.3–45.2)
≤ 149 min	2,486	56.3	(54.8–57.7)
**Sexual orientation**			
Heterosexual	3,694	88.8	(87.8–89.7)
Homosexual	71	1.7	(1.5–2.1)
Bisexual	214	5.1	(4.5–5.9)
Unsure	157	3.8	(3.2–4.4)
Other	23	0.6	(0.4–0.8)
**Offspring**			
With children	68	1.4	(1.1–1.7)
No children	4,969	98.6	(98.3–98.9)
**Number of sex partners in the last year**			
None	1,697	40.8	(39.3–42.3)
1 or 2	2,274	54.7	(53.2–56.2)
3 or more	188	4.5	(3.9–5.2)
**Civil status**			
Single	4,964	98.6	(98.2–98.8)
Married	61	1.2	(0.9–1.6)
Civil union agreement	7	0.1	(0.1–0.3)
Divorced	4	0.1	(0.0–0.2)
Widowed	1	0.0	(0.0–0.1)

*n, Number of participants; CI, Confidence Interval; SD, Standard Deviation*.

Univariate and multivariate logistic regression models were performed in three sequential steps: (1) Unadjusted models: all variables were assessed to determine if they were associated with each of the five outcomes: depression, anxiety, stress, insomnia, and suicidality. Those variables that had a univariable association (*p*-value ≤ 0.05), were selected to be included in the next step. (2) All variables were organized according to the following domains: personal, family, university, and SARS-CoV-2 related variables. For each domain, we conducted a multivariate logistic model, and those variables that had an association (*p*-value ≤ 0.05), were selected to be included in the final multivariate model. (3) The final model included all the variables associated with the outcomes in step 2. For steps 2 and 3, we included the variables sex and age and respective covariates in each model. In the case of independent variables reflecting a degree of intensity or growth, we conducted the Walt test to decide if they were included in each successive model. All statistical analyses were performed using Stata 15.

## Results

### Sample Description

The main characteristics of the sample are presented in Table 1. A total of 5,037 students answered the survey—the global response rate of 63.5%. Most of the students were females (70.4%) and freshmen students (25.2%). The mean age increased by academic year, starting from 19.6 (SD = 2.9) years old in the 1st academic year to 24.0 (SD = 1.9) years old in the 5th or higher academic year. Survey respondents were distributed over 22 different courses. Most students were Chilean (96.5%), Non-Indigenous (98.1%), and only studied (87.5%). Regarding health history, 19.4% had a chronic illness, 35.4% had a diagnosis of an anxiety disorder, and 32.7% had ADHD and depression (27.9%). A 45.4% of students had attended psychotherapy, and 35.7 had used medication for a psychiatric condition. A 43.7% practiced regular physical activity. Most of the sample referred to being heterosexual (88.8%), single (98.6%) with no children (98.6%). A 4.5% of students reported having had three or more sexual partners in the last year. Finally, substance use was highly prevalent, with 54.6% of students reporting alcohol use in the last month. Risk for alcohol use disorder was found in 18.1% of students, and the risk for cannabis use disorder was found in 0.5% of students (see Table 2).

**Table 2:** Substance use prevalence.

**Substance use time period**		**Total**		**Females**		**Males**	
	**Class year**	***n***	**% (95% CI)**	***n***	**% (95% CI)**	***n***	**% (95% CI)**
Lifetime tobacco use	1st	623	62.2 (59.2–65.2)	463	62.8 (59.3–66.2)	160	60.6 (54.6–66.3)
	2nd	525	67.4 (64.0–70.6)	376	67.6 (63.6–71.4)	149	66.8 (60.4–72.7)
	3rd	588	68.1 (64.9–71.2)	421	67.4 (63.6–70.9)	167	70.2 (64.1–75.7)
	4th	560	69.1 (65.9–72.2)	404	70.0 (66.0–73.5)	156	67.2 (60.9–73.0)
	5th +	528	71.3 (67.9–74.4)	331	70.0 (65.7–73.9)	197	73.5 (67.9–78.4)
	Total	2,824	67.3 (65.9–68.7)	1,995	67.2 (65.5–68.9)	829	67.7 (65.0–70.2)
Last month tobacco use	1st	180	18.0 (15.7–20.5)	127	17.2 (14.7–20.1)	53	20.1 (15.7–25.4)
	2nd	160	20.5 (17.8–23.5)	112	20.1 (17.0–23.7)	48	21.5 (16.6–27.4)
	3rd	198	22.9 (20.3–25.9)	142	22.7 (19.6–26.2)	56	23.5 (18.6–29.4)
	4th	173	21.4 (18.7–24.3)	124	21.5 (18.3–25.0)	49	21.1 (16.3–26.9)
	5th +	138	18.6 (16.0–21.6)	82	17.3 (14.2–21.0)	56	20.9 (16.4–26.2)
	Total	849	20.2 (19.1–21.5)	587	19.8 (18.4–21.2)	262	21.4 (19.2–23.8)
Last week tobacco use	1st	145	14.5 (12.4–16.8)	102	13.8 (11.5–16.5)	43	16.3 (12.3–21.3)
	2nd	125	16.0 (13.6–18.8)	94	16.9 (14.0–20.3)	31	13.9 (9.9–19.1)
	3rd	170	19.7 (17.2–22.4)	123	19.7 (16.7–23.0)	47	19.7 (15.2–25.3)
	4th	151	18.6 (16.1–21.5)	108	18.7 (15.7–22.1)	43	18.5 (14.0–24.1)
	5th +	114	15.4 (13.0–18.2)	65	13.7 (10.9–17.2)	49	18.3 (14.1–23.4)
	Total	705	16.8 (15.7–18.0)	492	16.6 (15.3–18.0)	213	17.4 (15.4–19.6)
Risk of tobacco use disorder	1st	6	0.5 (0.2–1.0)	5	0.5 (0.2–1.3)	1	0.3 (0.0–2.0)
	2nd	6	0.6 (0.2–1.4)	4	0.6 (0.2–1.6)	2	0.7 (0.2–2.8)
	3rd	15	1.5 (0.9–2.5)	9	1.3 (0.7–2.4)	6	2.2 (1.0–4.7)
	4th	10	1.1 (0.6–2.0)	7	1.0 (0.5–2.2)	3	1.1 (0.4–3.5)
	5th +	10	1.1 (0.6–2.1)	7	1.3 (0.6–2.6)	3	0.9 (0.3–2.9)
	Total	47	0.9 (0.7–1.2)	32	0.9 (0.6–1.3)	15	1.0 (0.6–1.7)
Lifetime vaping use	1st	384	38.4 (35.4–41.4)	256	34.7 (31.4–38.2)	128	48.5 (42.5–54.5)
	2nd	271	34.8 (31.5–38.2)	183	32.9 (29.1–36.9)	88	39.5 (33.3–46.0)
	3rd	299	34.6 (31.5–37.9)	189	30.2 (26.8–34.0)	110	46.2 (40.0–52.6)
	4th	277	34.2 (31.0–37.5)	182	31.5 (27.8–35.4)	95	40.9 (34.8–47.4)
	5th +	267	36.0 (32.7–39.6)	137	29.0 (25.1–33.2)	130	48.5 (42.6–54.5)
	Total	1498	35.7 (34.3–37.2)	947	31.9 (30.2–33.6)	551	45.0 (42.2–47.8)
Last month vaping use	1st	28	2.8 (1.9–4.0)	18	2.4 (1.5–3.8)	10	3.8 (2.0–6.9)
	2nd	19	2.4 (1.6–3.8)	10	1.8 (1.0–3.3)	9	4.0 (2.1–7.6)
	3rd	16	1.9 (1.1–3.0)	8	1.3 (0.6–2.5)	8	3.4 (1.7–6.6)
	4th	16	2.0 (1.2–3.2)	9	1.6 (0.8–3.0)	7	3.0 (1.4–6.2)
	5th +	17	2.3 (1.4–3.7)	7	1.5 (0.7–3.1)	10	3.7 (2.0–6.8)
	Total	96	2.3 (1.9–2.8)	52	1.8 (1.3–2.3)	44	3.6 (2.7–4.8)
Last week vaping use	1st	17	1.7 (1.1–2.7)	12	1.6 (0.9–2.8)	5	1.9 (0.8–4.5)
	2nd	15	1.9 (1.2–3.2)	8	1.4 (0.7–2.9)	7	3.1 (1.5–6.4)
	3rd	14	1.6 (1.0–2.7)	7	1.1 (0.5–2.3)	7	2.9 (1.4–6.1)
	4th	10	1.2 (0.7–2.3)	4	0.7 (0.3–1.8)	6	2.6 (1.2–5.6)
	5th +	9	1.2 (0.6–2.3)	2	0.4 (0.1–1.7)	7	2.6 (1.2–5.4)
	Total	65	1.5 (1.2–2.0)	33	1.1 (0.8–1.6)	32	2.6 (1.9–3.7)
Lifetime alcohol use	1st	753	75.1 (72.4–77.7)	540	73.2 (69.9–76.2)	213	80.7 (75.5–85.0)
	2nd	612	78.3 (75.2–81.0)	440	78.7 (75.1–81.9)	172	77.1 (71.2–82.2)
	3rd	722	83.6 (80.9–85.9)	509	81.3 (78.1–84.2)	213	89.5 (84.9–92.8)
	4th	706	87.2 (84.7–89.3)	497	86.0 (82.9–88.6)	209	90.1 (85.5–93.3)
	5th +	641	86.4 (83.7–88.7)	400	84.6 (81.0–87.6)	241	89.6 (85.3–92.7)
	Total	3434	81.8 (80.6–82.9)	2386	80.2 (78.8–81.6)	1048	85.5 (83.4–87.3)
Last month alcohol use	1st	478	47.7 (44.6–50.8)	323	43.8 (40.2–47.4)	155	58.7 (52.7–64.5)
	2nd	392	50.1 (46.6–53.7)	273	48.8 (44.7–53.0)	119	53.4 (46.8–59.8)
	3rd	478	55.3 (52.0–58.6)	328	52.4 (48.5–56.3)	150	63.0 (56.7–68.9)
	4th	489	60.4 (57.0–63.7)	335	58.0 (53.9–61.9)	154	66.4 (60.1–72.2)
	5th +	458	61.7 (58.2–65.2)	260	55.0 (50.5–59.4)	198	73.6 (68.0–78.5)
	Total	2295	54.6 (53.1–56.1)	1519	51.1 (49.3–52.9)	776	63.3 (60.6–66.0)
Last week alcohol use	1st	134	13.4 (11.4–15.6)	73	9.9 (7.9–12.3)	61	23.1 (18.4–28.6)
	2nd	114	14.6 (12.3–17.2)	71	12.7 (10.2–15.7)	43	19.3 (14.6–25.0)
	3rd	147	17.0 (14.7–19.7)	82	13.1 (10.7–16.0)	65	27.3 (22.0–33.3)
	4th	152	18.8 (16.2–21.6)	89	15.4 (12.7–18.6)	63	27.2 (21.8–33.2)
	5th +	149	20.1 (17.4–23.1)	55	11.6 (9.0–14.8)	94	34.9 (29.5–40.8)
	Total	696	16.6 (15.5–17.7)	370	12.4 (11.3–13.7)	326	26.6 (24.2–29.1)
Risk of alcohol use disorder	1st	171	13.5 (11.7–15.5)	109	11.8 (10.0–14.1)	62	17.9 (14.2–22.3)
	2nd	166	17.3 (15.0–19.8)	119	17.5 (14.8–20.6)	47	16.7 (12.7–21.5)
	3rd	216	21.8 (19.3–24.5)	131	18.3 (15.7–21.3)	85	30.7 (25.5–36.4)
	4th	214	22.9 (20.3–25.7)	137	20.4 (17.5–23.6)	77	29.1 (23.9–34.8)
	5th +	196	22.3 (19.6–25.1)	106	19.0 (15.9–22.4)	90	28.0 (23.4–33.2)
	Total	963	18.1 (17.0–19.1)	602	17.0 (15.8–18.2)	361	24.2 (22.1–26.5)
Lifetime cannabis use	1st	417	41.7 (38.6–44.7)	291	39.5 (36.0–43.1)	126	47.7 (41.8–53.8)
	2nd	379	48.5 (45.0–52.0)	265	47.7 (43.5–51.8)	113	50.7 (44.1–57.2)
	3rd	510	59.1 (55.8–62.3)	346	55.4 (51.4–59.2)	164	68.9 (62.7–74.5)
	4th	512	63.2 (59.8–66.5)	350	60.6 (56.5–64.5)	162	69.8 (63.6–75.4)
	5th +	514	69.4 (65.9–72.6)	300	63.4 (59.0–67.6)	214	79.9 (74.6–84.2)
	Total	2331	55.6 (54.1–57.1)	1552	52.3 (50.5–54.1)	779	63.6 (60.9–66.2)
Last month cannabis use	1st	44	4.4 (3.3–5.9)	22	3.0 (19.7–4.5)	22	8.3 (5.5–12.3)
	2nd	53	6.8 (5.2–8.8)	30	5.4 (3.8–7.6)	23	10.3 (6.9–15.1)
	3rd	72	8.3 (6.7–10.4)	37	5.9 (4.3–8.1)	35	14.7 (10.7–19.8)
	4th	74	9.1 (7.3–11.3)	47	8.1 (6.2–10.7)	27	11.6 (8.1–16.5)
	5th +	104	14.0 (11.7–16.7)	45	9.5 (7.2–12.5)	59	22.0 (17.4–27.4)
	Total	347	8.3 (7.5–9.1)	181	6.1 (5.3–7.0)	166	13.6 (11.7–15.6)
Last week cannabis use	1st	23	2.3 (1.5–3.4)	7	0.9 (0.5–2.0)	16	6.1 (3.7–9.7)
	2nd	28	3.6 (2.5–5.2)	14	2.5 (1.5–4.2)	14	6.3 (3.7–10.3)
	3rd	52	6.0 (4.6–7.8)	26	4.2 (2.8–6.0)	26	10.9 (7.5–15.6)
	4th	41	5.1 (3.7–6.8)	20	3.5 (2.2–5.3)	21	9.1 (6.0–13.5)
	5th +	63	8.5 (6.7–10.7)	24	5.1 (3.4–7.5)	39	14.6 (10.8–19.3)
	Total	207	4.9 (4.3–5.6)	91	3.1 (2.5–3.8)	116	9.5 (8.0–11.2)
Risk of cannabis use disorder	1st	5	0.4 (0.2–0.9)	1	0.1 (0.0–0.8)	4	1.2 (0.4–3.0)
	2nd	3	0.3 (0.1–1.0)	0	–	3	1.1 (0.3–3.3)
	3rd	7	0.7 (0.3–1.5)	2	0.3 (0.1–1.1)	5	1.8 (0.8–4.3)
	4th	5	0.5 (0.2–1.3)	1	0.1 (0.0–1.1)	4	1.5 (0.6–4.0)
	5th +	7	0.8 (0.4–1.7)	2	0.4 (0.1–1.4)	5	1.6 (0.6–3.7)
	Total	27	0.5 (0.3–0.7)	6	0.2 (0.1–0.4)	21	1.4 (0.9–2.2)
Lifetime tranquilizers use	1st	205	20.5 (18.1–23.1)	159	21.6 (18.8–24.7)	46	17.4 (13.3–22.5)
	2nd	195	25.0 (22.1–28.2)	152	27.3 (23.8–31.2)	43	19.3 (14.6–25.0)
	3rd	232	26.9 (24.0–29.9)	182	29.1 (25.7–32.8)	50	21.0 (16.3–26.7)
	4th	186	23.0 (20.2–26.0)	148	25.6 (22.2–29.3)	38	16.4 (12.2–21.7)
	5th +	227	30.6 (27.4–34.1)	161	34.1 (29.9–38.4)	66	24.6 (19.8–30.1)
	Total	1045	24.9 (23.6–26.3)	802	27.0 (25.4–28.6)	243	19.8 (17.7–2.2)
Last month tranquilizers use	1st	76	7.6 (6.1–9.4)	56	7.6 (5.9–9.7)	20	7.6 (4.9–11.5)
	2nd	84	10.8 (8.8–13.2)	64	11.5 (9.1–14.4)	20	9.0 (5.9–13.5)
	3rd	81	9.4 (7.6–11.5)	62	9.9 (7.8–12.5)	19	8.0 (5.1–12.2)
	4th	59	7.3 (5.7–9.3)	52	9.0 (6.9–11.6)	7	3.0 (1.4–6.2)
	5th +	63	8.5 (6.7–10.7)	48	10.1 (7.7–13.2)	15	5.6 (3.4–9.1)
	Total	363	8.7 (7.8–9.5)	282	9.5 (8.5–10.6)	81	6.6 (5.3–8.1)
Last week tranquilizers use	1st	53	5.3 (4.1–6.9)	42	5.7 (4.2–7.6)	11	4.2 (2.3–7.4)
	2nd	56	7.2 (5.6–9.2)	45	8.1 (6.1–10.7)	11	4.9 (2.7–8.7)
	3rd	61	7.1 (5.5–9.0)	48	7.7 (5.8–10.0)	13	5.5 (3.2–9.2)
	4th	42	5.2 (3.9–6.9)	38	6.6 (4.8–8.9)	4	1.7 (0.6–4.5)
	5th +	47	6.3 (4.8–8.3)	37	7.8 (5.7–10.6)	10	3.7 (2.0–6.8)
	Total	259	6.2 (5.5–6.9)	210	7.1 (6.2–8.1)	49	4.0 (3.0–5.3)
Risk of tranquilizers use disorder	1st	1	0.1 (0.0–0.6)	1	0.1 (0.0–0.8)	0	–
	2nd	9	0.9 (0.5–1.8)	6	0.9 (0.4–2.0)	3	1.1 (0.3–3.3)
	3rd	10	1.0 (0.5–1.9)	6	0.8 (0.4–1.9)	4	1.4 (0.5–3.8)
	4th	4	0.4 (0.2–1.1)	2	0.3 (0.1–1.2)	2	0.8 (0.2–3.0)
	5th +	4	0.5 (0.2–1.2)	4	0.7 (0.3–1.9)	0	–
	Total	28	0.5 (0.4–0.8)	19	0.5 (0.3–0.8)	9	0.6 (0.3–1.2)
Lifetime nootropics use	1st	257	25.7 (23.1–28.5)	182	24.7 (21.7–27.9)	75	28.4 (23.3–34.2)
	2nd	234	30.0 (26.9–33.4)	168	30.2 (26.5–34.2)	66	29.6 (24.0–35.9)
	3rd	279	32.3 (29.3–35.5)	190	30.4 (26.9–34.1)	89	37.4 (31.5–43.7)
	4th	262	32.4 (29.2–35.7)	185	32.0 (28.3–35.9)	77	33.2 (27.4–39.5)
	5th +	254	34.3 (29.2–37.8)	150	31.7 (27.7–36.1)	104	31.7 (27.7–36.1)
	Total	1286	30.7 (29.3–32.1)	875	29.5 (27.9–31.1)	411	33.6 (31.0–36.3)
Last month nootropics use	1st	79	7.9 (6.4–9.7)	58	7.9 (6.1–10.0)	21	8.0 (5.2–11.9)
	2nd	88	11.3 (9.3–13.7)	67	12.1 (9.6–15.0)	21	9.4 (6.2–14.0)
	3rd	78	9.0 (7.3–11.1)	53	8.5 (6.5–10.9)	25	10.5 (7.2–15.1)
	4th	66	8.1 (6.5–10.2)	50	8.7 (6.6–11.2)	16	6.9 (4.3–11.0)
	5th +	60	8.1 (6.3–10.3)	38	8.0 (5.9–10.9)	22	8.2 (5.5–12.2)
	Total	371	8.8 (8.0–9.7)	266	9.0 (8.0–10.0)	105	8.6 (7.1–10.3)
Last week nootropics use	1st	68	6.8 (5.4–8.5)	50	6.8 (5.2–8.8)	18	6.8 (4.3–10.6)
	2nd	72	9.2 (7.4–11.5)	55	9.9 (7.7–12.7)	17	7.6 (4.8–11.9)
	3rd	61	7.1 (5.5–9.0)	40	6.4 (4.7–8.6)	21	8.8 (5.8–13.2)
	4th	54	6.7 (5.1–8.6)	41	7.1 (5.3–9.5)	13	5.6 (3.3–9.4)
	5th +	51	6.9 (5.3–8.9)	32	6.8 (4.8–9.4)	19	7.1 (4.6–10.9)
	Total	306	7.3 (6.5–8.1)	218	7.3 (6.5–8.3)	88	7.2 (5.9–8.8)
Risk of nootropics use disorder	1st	7	0.6 (0.3–1.2)	6	0.7 (0.3–1.4)	1	0.3 (0.0–2.0)
	2nd	9	0.9 (0.5–1.8)	8	1.2 (0.6–2.3)	1	0.4 (0.0–2.5)
	3rd	5	0.5 (0.2–1.2)	1	0.1 (0.0–1.0)	4	1.4 (0.5–3.8)
	4th	5	0.5 (0.2–1.3)	4	0.6 (0.2–1.6)	1	0.4 (0.1–2.6)
	5th +	5	0.6 (0.2–1.4)	2	0.4 (0.1–1.4)	3	0.9 (0.3–2.9)
	Total	31	0.6 (0.4–0.8)	21	0.6 (0.4–0.9)	10	0.7 (0.4–1.2)

*Risk of vaping use disorder was not included because it had no observations; –, no observations in the category; n, number of participants; CI, Confidence Interval*.

In the case of the parents' educational level, most completed university studies (mothers, 55.8% and fathers 45.7%). A 39.1% had a family history of psychiatric disorders, and 11.4% had a family member who committed suicide. A third of students reported that they had dysfunctional families according to the APGAR (28.9%) (see Table 3).

**Table 3:** Family variables.

**Family variables**	***n***	**%**	**(95% CI)**
**Mother's educational level**			
Incomplete elementary school	14	0.3	(0.2–0.5)
Complete elementary school and incomplete high school	54	1.1	(0.9–1.5)
Complete high school and incomplete university	1,182	24.5	(23.3–25.7)
Complete university and incomplete postgraduate	2,691	55.8	(54.3–57.1)
Complete postgraduate	858	17.8	(16.7–18.9)
**Father's educational level**			
Incomplete elementary school	9	0.2	(0.1–0.4)
Complete elementary school and incomplete high school	56	1.2	(0.9–1.5)
Complete high school and incomplete university	934	19.4	(18.3–20.5)
Complete university and incomplete postgraduate	2,207	45.7	(44.3–47.1)
Complete postgraduate	1,522	31.5	(30.2–32.9)
**Family history of mental health problems**			
Psychiatric disorders	1,731	39.1	(37.7–40.6)
Suicide	504	11.4	(10.5–12.4)
Alcohol abuse or dependence	1,300	29.4	(28.1–30.7)
Drug abuse or dependence	510	11.5	(10.6–12.5)
**Family functioning (APGAR)**			
Highly functional	3,116	71.2	(69.8–72.4)
Moderate dysfunctional	798	18.2	(17.1–19.4)
Severe dysfunctional	467	10.7	(9.8–11.6)

*n, Number of participants; CI, Confidence Interval*.

For most students, their parents paid for their education (89.2%). The mean commuting time was 52.1 min (SD = 35.9), and 17.8% of students took more than 88 min from home to the university. A 35.5% of students reported having had failed a subject. A third of students had felt to be excluded from social gatherings in the university, and 9.5% had experienced psychological victimization. The lowest score on the psychological sense of university belonging was in Year 1 (85.6, SD = 0.4) and went up the following years but with no special difference between Year 2 and Year 5 or more. A 15.4% of students had a score higher than 1SD over average on the psychological sense of belonging (see Table 4).

**Table 4:** University variables.

**University variables**	***n***	**%**	**(95% CI)**
**Academic year**			
1st	1,268	25.2	(24.0–26.4)
2nd	961	19.1	(18.0–20.2)
3rd	992	19.7	(18.6–20.8)
4th	936	18.6	(17.5–19.7)
5th or higher	880	17.5	(16.4–18.5)
**Source of financing**			
Funding from parents	4,307	89.2	(88.3–90.1)
Credit/loan	1,623	33.6	(32.3–35.0)
Scholarship	1,539	31.9	(30.6–33.2)
Self-funded	250	5.2	(4.6–5.8)
Other means	224	4.6	(4.1–5.3)
**Commuting time university**			
>1SD	861	17.8	(16.8–18.9)
±1SD	3,317	68.7	(67.4–70.0)
< -1SD	649	13.5	(12.5–14.4)
**History of failing subjects**			
Yes	1,787	35.5	(34.2–36.8)
No	3,250	64.5	(63.2–65.8)
**Violence victimization**			
Physical	33	0.7	(0.5–1.0)
Psychological	422	9.5	(8.6–10.4)
Exclusion	1,318	29.6	(28.2–30.9)
Teasing	502	11.3	(10.4–12.2)
Ridiculization	514	11.5	(10.6–12.5)
**Psychological sense of university belonging**			
>1SD	693	15.4	(14.4–16.5)
±1SD	3,054	67.8	(66.5–69.2)
< -1SD	755	16.8	(15.7–17.9)

*Students may have more than one source of financing; n, Number of participants; CI, Confidence Interval*.

Regarding SARS-CoV-2 related variables, only 3.0% of students contracted SARS-CoV-2, and 29.5% of students reported that a family relative contracted SARS-CoV-2. Students had a higher sense of fear of having a friend or family member contracting SARS-CoV-2 than themselves. During lockdowns, most students lived with family, friends or roommates, 83.6% kept in touch with people, 78.5% had practiced physical exercise, 76.6% had been involved in recreational activities, 83.6% kept a daily routine, and 47.7% meditated or prayed (see Table 5).

**Table 5:** SARS-CoV-2 experiences variables.

**SARS-CoV-2 experiences**	***n***	**%**	**(95% CI)**
History of personal contagion of SARS-CoV-2	133	3.0	(2.6–3.6)
History of family contagion of SARS-CoV-2	1,289	29.5	(28.1–30.8)
**Fear of contracting SARS-CoV-2 by the students**			
Not at all	358	8.2	(7.4–9.0)
Slightly	840	19.2	(18.1–20.4)
Somewhat	1,510	34.5	(33.1–35.9)
Moderately	1,105	25.3	(24.0–26.6)
Extremely	563	12.9	(11.9–13.9)
**Fear of others contracting SARS-CoV-2**			
Not at all	46	1.1	(0.8–1.4)
Slightly	132	3.0	(2.5–3.6)
Somewhat	623	14.2	(13.2–15.3)
Moderately	1,493	34.1	(32.7–35.5)
Extremely	2,082	47.6	(46.1–49.1)
**Living condition during pandemic lockdowns**			
Living independently	80	1.8	(1.5–2.3)
Living with family, friends or roommates	4,296	98.2	(97.7–98.5)
**Frequency of social contact during lockdowns**			
Never	711	16.4	(15.3–17.5)
1–2 days a week	526	12.1	(11.2–44.4)
3–4 days a week	998	23.0	(21.8–24.3)
5–6 days a week	1,045	24.1	(22.8–25.4)
Everyday	1,061	24.4	(23.2–25.7)
**Frequency of physical exercising during lockdowns**			
Never	934	21.5	(20.3–22.8)
1–2 days a week	1,217	28.0	(26.7–29.4)
3–4 days a week	1,253	28.9	(27.5–30.2)
5–6 days a week	791	18.2	(17.1–19.4)
Everyday	146	3.4	(2.9–3.9)
**Frequency of recreational activities during lockdowns**			
Never	1,015	23.4	(22.1–24.7)
1–2 days a week	1,740	40.1	(38.6–41.5)
3–4 days a week	896	20.6	(19.5–21.9)
5–6 days a week	341	7.9	(7.1–8.7)
Everyday	349	8.0	(7.3–8.9)
**Keeping a routine during lockdowns**			
Never	711	16.4	(15.3–17.5)
1–2 days a week	526	12.1	(11.2–13.1)
3–4 days a week	998	23.0	(21.8–24.3)
5–6 days a week	1,045	24.1	(22.8–25.4)
Everyday	1,061	24.4	(23.2–25.7)
**Frequency of meditation or praying during lockdowns**			
Never	2,269	52.3	(50.8–53.8)
1–2 days a week	1,146	26.4	(25.1–27.7)
3–4 days a week	387	8.9	(8.1–9.8)
5–6 days a week	181	4.2	(3.6–4.8)
Everyday	358	8.3	(7.5–9.1)

*n, Number of participants; CI, Confidence Interval*.

### Mental Health Problems

Depression symptoms were reported in 37.1% of students, and among females reached 38.7%, higher than in males (33.2%). Anxiety symptoms were reported in 37.9% of students (females, 42.5%; males, 26.9%). Stress symptoms were reported in 54.6% of students (females, 58.5%; males, 45.1%). Insomnia was reported in 32.5% of students (females, 33.9%; males, 29.2%). Suicide risk was reported in 20.4% of students, higher among females (20.9%) than in males (19.2%) (see Table 6).

**Table 6:** Psychological symptoms (DASS-21), insomnia, and suicide risk by sex and grade.

**Variable**		**Total**		**Females**		**Males**	
	**Grade**	***n***	**% (95% CI)**	***n***	**% (95% CI)**	***n***	**% (95% CI)**
**Depression**							
	1st	395	39.1 (36.1–42.1)	303	40.7 (37.2–44.2)	92	34.6 (29.1–40.1)
	2nd	323	41.1 (37.7–44.6)	254	45.3 (41.2–49.4)	69	30.7 (25.0–37.0)
	3rd	337	38.6 (35.5–41.2)	252	39.8 (36.1–43.7)	85	35.6 (29.8–41.8)
	4th	274	33.6 (30.4–36.9)	195	33.6 (29.9–37.6)	79	33.5 (27.7–39.7)
	5th +	240	32.0 (28.8–35.5)	154	32.3 (28.2–36.6)	86	31.6 (26.4–37.4)
	Total	1,569	37.1 (35.6–38.5)	1,158	38.7 (36.9–40.4)	411	33.2 (30.6–35.9)
**Anxiety**							
	1st	399	39.5 (36.5–42.5)	333	44.7 (41.2–48.3)	66	24.8 (20.0–30.4)
	2nd	324	41.2 (37.8–44.7)	258	46.0 (41.9–50.1)	66	29.3 (23.8–35.6)
	3rd	341	39.1 (35.9–42.4)	262	41.4 (37.6–45.3)	79	33.1 (27.4–39.3)
	4th	301	36.9 (33.6–40.3)	245	42.2 (38.3–46.3)	56	23.7 (18.7–29.6)
	5th +	241	32.2 (28.9–35.6)	175	36.7 (32.5–41.1)	66	24.3 (19.5–29.7)
	Total	1,606	37.9 (36.5–39.4)	1,273	42.5 (40.7–44.3)	333	26.9 (24.5–29.4)
**Stress**							
	1st	561	55.5 (52.4–58.5)	441	59.2 (55.6–62.7)	120	45.1 (39.2–51.1)
	2nd	445	56.6 (53.1–60.0)	345	61.5 (57.4–65.4)	100	44.4 (38.1–51.0)
	3rd	480	55.5 (51.7–58.3)	366	57.8 (53.9–61.6)	114	47.7 (41.4–54.0)
	4th	434	53.2 (49.8–56.6)	329	56.7 (52.7–60.7)	105	44.5 (38.3–50.9)
	5th +	390	52.1 (48.5–55.6)	271	56.8 (52.3–61.2)	119	43.8 (38.0–49.7)
	Total	2,310	54.6 (53.1–56.1)	1,752	58.5 (56.7–60.2)	558	45.1 (42.3–47.9)
**Insomnia**							
	1st	330	32.9 (30.0–35.8)	257	34.8 (31.4–38.3)	73	27.5 (22.5–33.2)
	2nd	262	33.4 (30.2–36.8)	194	34.6 (30.8–38.7)	68	30.4 (24.7–36.7)
	3rd	300	34.6 (31.5–37.8)	225	35.7 (32.1–39.5)	75	31.5 (25.9–37.7)
	4th	261	32.1 (29.0–35.4)	190	32.9 (29.2–36.8)	71	30.3 (24.8–36.5)
	5th +	216	29.1 (25.9–32.4)	144	30.4 (26.5–32.3)	72	26.7 (21.7–32.3)
	Total	1,369	32.5 (31.1–33.9)	1,010	33.9 (32.2–35.6)	359	29.2 (26.7–31.8)
**Suicide Risk**							
	1st	201	20.1 (17.7–22.7)	160	21.7 (18.9–24.8)	41	15.5 (11.6–20.4)
	2nd	161	20.5 (17.8–23.5)	123	22.0 (18.7–25.6)	38	17.0 {12.6–22.5)
	3rd	196	22.6 (19.9–25.5)	146	23.2 (20.0–26.6)	50	21.0 (16.3–26.7)
	4th	150	18.5 (16.0–21.3)	101	17.5 (14.6–20.8)	49	21.1 (16.3–26.9)
	5th +	149	20.1 (17.3–23.1)	91	19.2 (15.9–23.0)	58	21.6 (17.0–26.9)
	Total	857	20.4 (19.2–21.6)	621	20.9 (19.4–22.3)	236	19.2 (17.1–21.5)

*n, Number of participants; CI, Confidence Interval*.

### Associations

The univariable and multivariable models by domain are presented in the Supplementary Material. The final multivariate model is presented in Table 7.

**Table 7:** Final multivariable associations between risk and protective factors and the outcomes depression, anxiety, stress, insomnia, and suicide risk.

**Risk and protective factors**	**Depression**	**Anxiety**	**Stress**	**Insomnia**	**Suicide risk**
	**OR (95% CI) *p*-value**	**OR (95% CI) *p*-value**	**OR (95% CI) *p*-value**	**OR (95% CI) *p*-value**	**OR (95% CI) *p*-value**
**Personal domain**					
Sex (ref = Male)					
Female	1.03 (0.86–1.24) 0.741	1.54 (1.28–1.85) <0.001	1.39 (1.17–1.65) <0.001	1.07 (0.90–1.28) 0.414	0.97 (0.78–1.20) 0.784
Age	0.95 (0.91–1.00) 0.034	0.91 (0.86–0.95) <0.001	0.93 (0.90–0.97) <0.001	0.94 (0.91–0.97) <0.001	0.94 (0.90–0.98) 0.004
Occupational status (ref = Studying only)		*0.6812			*0.8191
Studying and breakt-time job	–	0.93 (0.72–1.20) 0.553	–	–	1.09 (0.82–1.44) 0.564
Studying and full-time job	–	0.73 (0.31–1.74) 0.474	–	–	1.15 (0.51–2.63) 0.733
History of chronic illness (ref = No)					
Yes	–	1.41 (1.17–1.69) <0.001	–	1.20 (1.00–1.43) 0.045	1.17 (0.94–1.45) 0.154
History of mental health disorders					
Depression	1.79 (1.49–2.15) <0.001	1.18 (0.97–1.44) 0.093	1.11 (0.92–1.35) 0.279	–	1.85 (1.50–2.28) <0.001
Bipolar disorder	1.58 (0.99–2.52) 0.053	–	–	–	1.75 (1.13–2.71) 0.013
Panic disorders	1.28 (1.06–1.56) 0.012	2.23 (1.83–2.70) <0.001	1.34 (1.09–1.64) 0.005	1.37 (1.14–1.65) 0.001	–
Anxiety disorders	1.40 (1.18–1.67) <0.001	1.92 (1.60–2.29) <0.001	1.98 (1.66–2.36) <0.001	1.24 (1.05–1.46) 0.011	1.37 (1.12–1.67) 0.002
Eating disorders	1.40 (1.09–1.79) 0.008	1.21 (0.94–1.56) 0.130	1.20 (0.93–1.56) 0.162	1.28 (1.02–1.61) 0.035	–
ADHD	–	–	–	1.33 (1.14–1.55) <0.001	–
History of mental health treatment					
Psychotherapy	–	–	1.16 (0.99–1.36) 0.068	–	1.21 (0.99–1.48) 0.064
Pharmacologic	–	0.84 (0.70–1.02) 0.075	–	–	–
Physical activity during the week (ref = ≤ 149 mins					
≥ 150 min	0.93 (0.75–1.15) 0.480	0.83 (0.66–1.03) 0.083	0.97 (0.84–1.12) 0.652	1.12 (0.91–1.37) 0.302	0.80 (0.61–1.03) 0.083
Sexual orientation (ref = Heterosexual)					
Homosexual	1.63 (0.90–2.95) 0.106	1.60 (0.89–2.90) 0.119	1.40 (0.77–2.55) 0.274	0.76 (0.43–1.34) 0.344	3.14 (1.77–5.57) <0.001
Bisexual	1.38 (0.99–1.92) 0.061	1.52 (1.09–2.13) 0.014	1.08 (0.76–1.53) 0.677	1.15 (0.84–1.57) 0.376	2.14 (1.53–2.98) <0.001
Unsure	1.58 (1.08–2.31) 0.017	1.44 (0.98–2.12) 0.061	1.38 (0.93–2.07) 0.111	1.04 (0.72–1.50) 0.851	2.45 (1.67–3.59) <0.001
Other	0.65 (0.24–1.75) 0.391	0.84 (0.31–2.31) 0.735	0.42 (0.16–1.10) 0.077	0.55 (0.21–1.44) 0.223	1.74 (0.64–4.71) 0.274
Number of sex breaktners in the last year (ref = none)		*0.1046	*0.1584		
1 or 2	–	1.15 (0.97–1.36) 0.088	1.15 (0.99–1.34) 0.070	–	–
3 or more	–	0.87 (0.58–1.29) 0.476	0.98 (0.67–1.42) 0.908	–	–
Substance use					
Risk of tobacco use disorder	–	2.46 (1.17–5.17) 0.017	2.29 (1.02–5.11) 0.044	–	–
Last month alcohol use	0.84 (0.72–0.98) 0.022	0.91 (0.77–1.06) 0.214	0.89 (0.77–1.04) 0.141	–	0.73 (0.61–0.89) 0.001
Risk of alcohol use disorder	–	–	–	1.29 (1.09–1.53) 0.003	1.60 (1.29–1.97) <0.001
Last month cannabis use	1.16 (0.88–1.54) 0.287	1.21 (0.91–1.63) 0.194	1.37 (1.03–1.82) 0.030	–	1.13 (0.82–1.54) 0.455
Last month tranquilizers use	1.57 (1.20–2.05) 0.001	1.43 (1.25–2.17) <0.001	1.73 (1.29–2.32) 0.001	3.28 (2.55–4.22) <0.001	1.23 (0.92–1.63) 0.158
Last month nootropics use	–	1.82 (1.39–2.37) <0.001	–	–	–
Risk of nootropics use disorder	–	–	4.56 (1.16–17.91) 0.030	–	–
**Family domain**					
Father's educational level (ref = Incomplete elementary school)		*0.0656	*0.1733		
Complete elementary school and incomplete high school	–	1.23 (0.17–8.66) 0.836	2.08 (0.30–14.55) 0.461	–	–
Complete high school and incomplete university	–	0.54 (0.09–3.38) 0.508	1.13 (0.18–6.92) 0.894	–	–
Complete university and incomplete postgraduate	–	0.49 (0.08–3.08) 0.448	1.02 (0.17–6.22) 0.984	–	–
Complete postgraduate	–	0.46 (0.07–2.91) 0.411	0.94 (0.15–5.78) 0.950	–	–
Family history of mental health problems					
Psychiatric disorders	1.08 (0.92–1.26) 0.378	1.22 (1.03–1.43) 0.017	1.15 (0.99–1.34) 0.062	1.07 (0.92–1.25) 0.365	1.18 (0.97–1.43) 0.094
Suicide	–	–	–	–	1.37 (1.06–1.78) 0.017
Alcohol abuse or dependence	1.05 (0.89–1.24) 0.588	1.10 (0.93–1.31) 0.265	–	1.01 (0.86–1.19) 0.881	1.11 (0.90–1.37) 0.318
Drug abuse or dependence	–	–	–	–	1.15 (0.88–1.51) 0.308
Family functioning (APGAR) (ref = Highly functional)	*0.0001	*0.0001	*0.0001	*0.0022	*0.0001
Moderate dysfunctional	2.04 (1.69–2.46) <0.001	1.58 (1.30–1.92) <0.001	1.69 (1.40–2.05) <0.001	1.23 (1.02–1.48) 0.030	2.17 (1.76–2.69) <0.001
Severe dysfunctional	3.44 (1.69–2.46) <0.001	1.98 (1.53–2.55) <0.001	2.65 (2.01–3.50) <0.001	1.47 (1.16–1.85) 0.001	3.64 (2.83–4.68) <0.001
**University domain**					
Academic year (ref = 1st)	*0.0210	*0.1704			
2nd	1.14 (0.90–1.44) 0.271	1.18 (0.93–1.49) 0.174	–	–	–
3rd	0.88 (0.69–1.13) 0.330	0.92 (0.72–1.18) 0.497	–	–	–
4th	0.79 (0.60–1.05) 0.101	1.05 (0.79–1.39) 0.735	–	–	–
5th or higher	0.70 (0.51–0.97) 0.033	0.86 (0.62–1.19) 0.366	–	–	–
Source of financing					
Self-funded	1.33 (0.94–1.90) 0.111	2.06 (1.40–3.05) <0.001	2.07 (1.39–3.08) <0.001	2.18 (1.58–3.02) <0.001	1.42 (0.96–2.09) 0.081
Commuting time university (ref = ±1SD)	*0.0833	*0.0278	*0.6062		*0.0092
>1SD	1.24 (1.02–1.51) 0.027	1.27 (1.04–1.55) 0.019	1.11 (0.91–1.35) 0.318	–	1.41 (1.13–1.76) 0.002
< -1SD	1.08 (0.87–1.35) 0.483	1.21 (0.97–1.51) 0.096	1.01 (0.82–1.24) 0.915	–	1.07 (0.81–1.41) 0.618
History of failing subjects (ref = No failed subjects)					
Failed subjects	1.05 (0.88–1.24) 0.598	–	1.03 (0.88–1.21) 0.679	1.25 (1.07–1.47) 0.005	1.12 (0.93–1.36) 0.234
Violence victimization					
Physical	–	2.26 (0.77–6.60) 0.136	–	1.81 (0.76–4.31) 0.181	1.71 (0.67–4.34) 0.258
Psychological	1.12 (0.85–1.47) 0.420	1.28 (0.97–1.69) 0.081	1.70 (1.28–2.27) <0.001	–	1.19 (0.90–1.57) 0.219
Exclusion	1.44 (1.21–1.71) <0.001	1.48 (1.24–1.77) <0.001	1.50 (1.26–1.78) <0.001	1.16 (0.97–1.37) 0.096	1.47 (1.21–1.79) <0.001
Teasing	1.25 (0.97–1.60) 0.081	1.36 (1.06–1.76) 0.017	–	1.18 (0.94–1.48) 0.162	–
Psychological sense of university belonging (ref = ±1SD)	*0.0001	*0.0004	*0.0001	*0.0030	*0.0001
>1SD	0.55 (0.43–0.70) <0.001	0.72 (0.57–0.91) 0.005	0.64 (0.52–0.78) <0.001	0.68 (0.55–0.85) 0.001	0.67 (0.49–0.92) 0.013
< -1SD	1.72 (1.39–2.12) <0.001	1.31 (1.06–1.63) 0.013	1.56 (1.24–1.95) <0.001	0.94 (0.77–1.15) 0.556	1.46 (1.17–1.82) 0.001
**SARS-CoV-2 experiences domain**					
History of family contagion of SARS-CoV-2 (ref = No)					
Yes	–	1.03 (0.88–1.22) 0.699	1.14 (0.98–1.34) 0.091	1.06 (0.91–1.24) 0.463	1.04 (0.86–1.26) 0.700
Fear to contracting SARS-CoV-2 by the students (ref = Not at all)	*0.0046	*0.0001	*0.0001	*0.0001	*0.0566
Slightly	1.06 (0.77–1.47) 0.712	1.13 (0.80–1.60) 0.485	1.26 (0.92–1.72) 0.143	1.14 (0.83–1.57) 0.425	0.68 (0.47–0.98) 0.041
Somewhat	0.84 (0.61–1.15) 0.275	1.31 (0.94–1.82) 0.114	1.51 (1.12–2.04) 0.007	1.09 (0.80–1.48) 0.598	0.62 (0.43–0.88) 0.007
Moderately	1.04 (0.75–1.46) 0.798	1.54 (1.09–2.19) 0.015	1.94 (1.41–2.68) <0.001	1.21 (0.88–1.68) 0.244	0.70 (0.50–1.01) 0.059
Extremely	1.35 (0.93–1.95) 0.115	2.29 (1.56–3.36) <0.001	2.84 (1.97–4.10) <0.001	1.97 (1.38–2.80) <0.001	0.81 (0.54–1.22) 0.322
Fear of others contracting SARS-CoV-2 (ref = Not at all)	*0.0001	*0.0001	*0.0455	*0.0001	*0.0129
Slightly	0.34 (0.14–0.79) 0.013	0.59 (0.24–1.48) 0.263	0.87 (0.38–1.99) 0.738	0.74 (0.33–1.70) 0.484	0.68 (0.28–1.67) 0.400
Somewhat	0.30 (0.14–0.65) 0.002	0.83 (0.37–1.86) 0.658	1.15 (0.54–2.42) 0.719	0.57 (0.27–1.20) 0.137	0.46 (0.21–1.03) 0.059
Moderately	0.42 (0.20–0.90) 0.025	0.94 (0.42–2.07) 0.870	1.23 (0.59–2.60) 0.578	0.78 (0.38–1.63) 0.513	0.60 (0.27–1.32) 0.203
Extremely	0.58 (0.27–1.24) 0.163	1.42 (0.64–3.14) 0.390	1.50 (0.71–3.16) 0.288	1.03 (0.50–2.15) 0.927	0.77 (0.35–1.69) 0.512
Frequency of social contact during lockdowns (ref = Never)	*0.3578	*0.1204	*0.7611		*0.1250
1–2 days a week	0.92 (0.65–1.29) 0.619	0.72 (0.51–1.02) 0.067	0.89 (0.63–1.26) 0.523	–	0.94 (0.65–1.37) 0.760
3–4 days a week	0.77 (0.53–1.11) 0.154	0.81 (0.56–1.17) 0.264	0.81 (0.56–1.18) 0.276	–	0.74 (0.49–1.11) 0.145
5–6 days a week	0.80 (0.53–1.22) 0.307	0.60 (0.39–0.92) 0.019	0.91 (0.60–1.39) 0.669	–	0.71 (0.44–1.16) 0.171
Everyday	0.84 (0.57–1.22) 0.359	0.81 (0.55–1.19) 0.228	0.84 (0.57–1.23) 0.373	–	1.02 (0.67–1.55) 0.941
Frequency of physical exercising during lockdowns (ref = Never)	*0.5872	*0.8000		*0.1476	*0.9061
1–2 days a week	0.86 (0.70–1.06) 0.147	0.89 (0.72–1.10) 0.290	–	1.02 (0.84–1.25) 0.823	1.00 (0.79–1.27) 1.000
3–4 days a week	0.85 (0.66–1.09) 0.195	0.96 (0.75–1.25) 0.782	–	0.79 (0.62–1.00) 0.053	0.92 (0.68–1.24) 0.584
5–6 days a week	0.82 (0.60–1.12) 0.215	0.92 (0.67–1.26) 0.599	–	0.81 (0.60–1.10) 0.175	0.89 (0.61–1.30) 0.552
Everyday	0.76 (0.45–1.27) 0.298	0.83 (0.50–1.37) 0.462	–	0.68 (0.41–1.11) 0.119	0.75 (0.40–1.41) 0.378
Frequency of recreational activities during lockdowns (ref = Never)	*0.0002		*0.0024		
1–2 days a week	0.76 (0.63–0.92) 0.005	–	0.86 (0.71–1.04) 0.127	–	–
3–4 days a week	0.71 (0.56–0.89) 0.003	–	0.71 (0.57–0.89) 0.003	–	–
5–6 days a week	0.57 (0.41–0.80) 0.001	–	0.78 (0.57–1.05) 0.097	–	–
Everyday	0.52 (0.38–0.73) <0.001	–	0.58 (0.43–0.78) <0.001	–	–
Keeping a routine during lockdowns (ref = Never)	*0.0001	*0.0103	*0.0030	*0.0001	*0.1229
1–2 days a week	0.93 (0.71–1.21) 0.583	0.79 (0.60–1.04) 0.099	0.73 (0.56–0.97) 0.027	0.70 (0.54–0.90) 0.006	0.96 (0.71–1.30) 0.775
3–4 days a week	0.64 (0.50–0.80) <0.001	0.76 (0.60–0.97) 0.028	0.79 (0.62–1.00) 0.054	0.59 (0.48–0.74) <0.001	0.79 (0.60–1.04) 0.092
5–6 days a week	0.53 (0.42–0.68) <0.001	0.66 (0.52–0.85) 0.001	0.63 (0.50–0.80) <0.001	0.44 (0.35–0.56) <0.001	0.71 (0.53–0.94) 0.017
Everyday	0.58 (0.46–0.74) <0.001	0.67 (0.52–0.86) 0.001	0.68 (0.54–0.87) 0.002	0.46 (0.36–0.57) <0.001	0.80 (0.60–1.06) 0.118
Frequency of meditation or praying during lockdowns (ref = Never)	*0.4292	*0.5960	*0.0444		*0.2271
1–2 days a week	0.91 (0.76–1.09) 0.319	0.99 (0.82–1.18) 0.877	1.00 (0.85–1.19) 0.956	–	1.10 (0.89–1.36) 0.391
3–4 days a week	1.02 (0.78–1.33) 0.905	1.22 (0.93–1.60) 0.143	1.33 (1.02–1.74) 0.033	–	1.31 (0.97–1.77) 0.080
5–6 days a week	0.88 (0.59–1.31) 0.516	0.95 (0.64–1.41) 0.806	0.76 (0.53–1.10) 0.141	–	0.80 (0.49–1.31) 0.370
Everyday	0.77 (0.57–1.04) 0.084	0.95 (0.71–1.28) 0.743	1.23 (0.93–1.61) 0.144	–	0.88 (0.61–1.27) 0.481

^*^Walt test; n, number of participants; CI, Confidence Interval.

*The following variables did not enter into the final multivariable models, so they were removed from the table to simplify the presentation of results: Nationality, Ethnicity, Offspring, Last month tobacco use, Risk of cannabis use disorder, Risk of tranquilizers use disorder, Mother's educational level, Source of financing (Funding from parents, Credit/Loan, Scholarship, Other means), Violence victimization (Ridiculization), History of personal contagion of SARS-CoV-2, Living condition during pandemic lockdowns*.

*The cells with the “–” symbol represent that the independent variable was not associated with the outcome in the previous analyses (univariable or multivariable by domains)*.

*The model was adjusted by sex and age*.

In the personal domain, females were more likely of suffering anxiety (OR 1.54; 1.28–1.85 95% CI) and stress symptoms (OR 1.39; 1.17–1.65 95% CI). Higher age reduced the odds for all outcomes. Having a history of chronic illness increased the odds for anxiety (OR 1.41; 1.17–1.69 95% CI) and insomnia (OR 1.20; 1.00–1.43 95% CI). Regarding previous diagnoses of mental health disorders, there was a positive association with several outcomes (see Table 7 for details). Regarding sexual orientation, homosexuals, bisexuals, and those students who reported to be unsure about their sexuality had higher odds of suicide risk. The consumption of different substances increased the odds of several outcomes, especially anxiety and stress (see Table 7 for details). On the other hand, last month alcohol use decreased the odds of depressive (OR 0.84; 0.72–0.98 95% CI). and suicide risk (OR 0.73; 0.61–0.89 95% CI).

In the family domain, family history of suicide increased the odds of suicide risk (OR 1.37; 1.06–1.78 95% CI), and family history of any psychiatric disorder increased the odds of anxiety symptoms (OR 1.22; 1.03–1.43 95% CI). Additionally, students who reported having a dysfunctional family were less likely to report symptoms consistent with each of the mental health outcomes. In the university domain, students who were in the 5th or higher academic year were less likely of suffering depressive symptoms (OR 0.70; 0.51–0.97 95% CI). In addition, students who had to pay for college by themselves were more likely of suffering anxiety symptoms (OR 2.06; 1.40–3.05 95% CI), stress (OR 2.07; 1.39–3.08 95% CI), and insomnia (OR 2.18; 1.58–3.02 95% CI). Students who take longer time to get the university were more likely to had depressive (OR 1.24; 1.02–1.51 95% CI) and anxiety symptoms (OR 1.27; 1.04–1.55 95% CI) and suicide risk (OR 1.41; 1.13–1.76 95% CI). Students who had psychological experiences of abuse at college had higher odds of stress (OR 1.70; 1.28–2.27 95% CI), students who had exclusion experiences at university had higher odds for depressive (OR 1.44; 1.21–1.71 95% CI) and anxiety symptoms (OR 1.48; 1.24–1.77 95% CI), stress (OR 1.50; 1.26–1.78 95% CI), and suicide risk (OR 1.47; 1.21–1.79 95% CI), and students who had teasing experiences had higher odds for anxiety symptoms (OR 1.36; 1.06–1.76 95% CI). Finally, students with a higher sense of university belonging were less likely to report symptoms consistent with each of the mental health outcomes.

In the case of pandemic domain variables, the higher the fear of contracting SARS-CoV-2 by students themselves or others, the greater the odds for depressive and anxiety symptoms, stress, and insomnia. Students who were involved in recreational activities during lockdowns decreased the odds of depressive symptoms and stress. Finally, students who kept a routine during lockdowns decreased the odds of depressive and anxiety symptoms, stress, and insomnia (see Table 7).

## Discussion

This is one of the first studies exploring mental health problems and their associated factors during the SARS-CoV-2 pandemic in Latin America. Mental health problems such as depression, anxiety, insomnia, and substance use are high among undergraduate students for all years of their careers. We also found a high prevalence of suicide risk (20.4%). A large proportion of students had a history of mental health problems. At the moment of the survey, 41.6% of this population was receiving psychological or psychiatric support. Finally, family history of psychiatric disorders (39.1%) and suicide (11.4%) were frequent among students. During the pandemic, very few students or their families had SARS-CoV-2. However, many students felt fear that themselves or any other family member or friend may contract SARS-CoV-2.

Few studies have explored the prevalence of mental health issues among undergraduate students in a SARS-CoV-2 context. A recent systematic review and meta-analysis included 16 articles exploring the prevalence of anxiety and depressive symptoms using valid and reliable instruments among university students during the pandemic. The pooled prevalence of anxiety symptoms was 31% (95% CI: 23–39%), and the pooled prevalence of depressive symptoms was 34%; both findings were similar to the ones found in our study (8). A large study conducted in France, surveying 69,054 students, found that suicidal thoughts prevalence was 11.4% (7,891 students), high level of perceived stress affected 24.7% (17,093 students), severe depression at 16.1% (11,133 students), and high level of anxiety at 27.5% (18,970 students) (18). In the United States, Wang et al. surveyed 2,031 students using the PHQ-9 and the GAD-7 and found a prevalence of 48.14% for depression, 38.48% for anxiety, and 18.04% for suicidal thoughts (9). Another study among 517 undergraduates in Spain found a high risk of suicide in 22.8% of students (43). These results are similar compared to our study. However, in Japan (44), the results seem to be different. For instance, depression was 11.7% (similar both in men and women), and suicidal ideation was 6.7%, also similar in men and women.

Even though our results are similar to other studies during the pandemic, the prevalence of mental health symptoms among Chilean undergraduates seems to have increased in recent years. No single cause can be attributed to this increase, but we can mention some potential contributing factors. In recent years, Chile has significantly reduced its economic growth (45); has experienced an unprecedented social outbreak (46), just before the pandemic; and has experienced the pandemic with the health restrictions that have accompanied it, such as the lockdowns (47). These events have been associated with reduced economic expectations, low job creation, and unemployment, especially for young people (48), all which have a clear detrimental impact on the population's mental health (47, 49–51). The context of mental health care where these events have been unfolded needs to be also considered. For instance, the treatment gap for mental disorders is high in Chile, reaching 38.5% among adults, and one-fifth of children or adolescents with a diagnosis receive any mental health service (52). Additionally, there is still an important stigma related to seeking health for mental problems (53–55). Nevertheless, a definitive explanation and the relative importance of the events presented above cannot be determined with the information available, and further research is needed.

Among the most urgent aspects found in our study was the high prevalence of suicide risk, which seem to be in the higher end range of the prevalence worldwide. Regarding suicide ideation, the global prevalence of suicide ideation has been stated in 10,6% among college students during pre-pandemic (4); however, and as we previously mentioned, some studies during the pandemic have found higher figures (9, 43). Even though the figures of suicide seem to be stable and even lower in the pandemic compared with the pre-pandemic times (56), our results show that undergraduate students have increased the psychological suffering during pandemic which stress the need for preventive measures incorporating the modifiable associated factors found in our study.

On the one hand, several factors were associated with an increased risk of having mental health issues. For example, being female, personal and family history of mental health disorders, tranquilizers use, having dysfunctional families, self-funding university, taking longer than average to get to the university campus, having exclusion experiences at university, and fear of themselves contracting SARS-CoV-2. These results are shared by other studies. For example, being female was also considered a risk factor for stress and anxiety (14, 18). Regarding family functioning, Shao et al., using the same instrument used in our study (57) found that the higher the score on family functionality, the better mental health, and that perceived good family support was a protective factor against poor mental health (58). Family functioning was negatively affected during the pandemic, especially due to changes in workload by parents, economic problems, changes in the daily routine due to lockdowns, and reduced social contact and mobility. Therefore, it was expected to be associated with mental health issues. Additionally, fear of contracting SARS-CoV-2 was also found in other studies. For instance, in Ecuador, fear of Covid-19 was a predictor of depression among university students, and another study found that increased levels of anxiety were associated with having a family member diagnosed with COVID-19 (59). Some studies in the general population have also reported severe stress responses such as post-traumatic stress disorder as a consequence of being exposed to a family affected with Covid-19 (60).

Regarding university experiences, victimization, such as social exclusion, was an important associated factor. In a study cross-cultural study, it was found that bullying victimization was associated with higher scores on DASS (61). On the other hand, to the best of our knowledge, our study is the only one that has explored the effect of the sense of belonging and its relationship with mental health during the pandemic. We found that a higher psychological sense of university was associated with reduced odds for all mental health outcomes. Pre-pandemic studies have shown that students' engagement in university life and relationships with peers and faculty members is fundamental for their wellbeing (62). Another study (63) showed that a sense of university belonging influenced mental health and academic outcomes and reduced depression, anxiety and stress symptoms among undergraduates (64). Potential interventions promoting university involvement and a sense of belonging may help to prevent mental health problems among this population and promote greater career satisfaction and success and community involvement in the future (65).

In the present study, we found that having a daily routine during lockdowns (sticking to a daily schedule) was another variable related to positive mental health. Few studies have assessed this association. For instance, one study conducted in a Spanish university (66) found that students who had a routine during the pandemic had a lower risk of mental health problems. Having a routine was one of the most recommended measures to prevent mental health problems at the beginning of the pandemic and quarantine measures proposed by several experts and institutions (67). Our results supported these recommendations.

We found mixed results on the association between substance use and mental health. On the one hand, the consumption of tobacco, cannabis, nootropics, and tranquilizers were associated with some mental health outcomes. These results were also supported by the findings among university students in Brazil (22). Tranquilizer use was associated with most of the mental health outcomes, and to our knowledge, no other study has explored this specific association among undergraduates during the pandemic. We only found one study in Mexico among the general population where they found that tranquilizer use increased the levels of stress and depressive symptomatology (68). On the other hand, we found that alcohol use was associated with a reduced risk for depressive symptoms and suicide risk. One recent study during the pandemic found that alcohol consumption (amount and frequency) increased as time progressed among university students (16). In addition, this study found that students with more symptoms of depression and anxiety reported had a greater increase in alcohol consumption. Therefore, our results may be seen as contradictory. However, we also found that the presence of alcohol social consequences such as having people criticizing your drinking (measured by CAGE and labeled as Risk of Alcohol Use Disorder), increased the odds for suicide risk. A recent study among female college students found that the quantity of alcohol consumed did not predict the onset of depression; however, experiencing alcohol consequences, regardless of consumption, did increase the risk of incident depression (69). We did not explore if alcohol use started recently or if this consumption was initiated long before the survey. We also do not know the longitudinal progression of alcohol use and depressive symptoms. The self-medication hypothesis says that individuals may use alcohol to reduce psychological distress, and we may have surveyed the students just in the time when their symptoms have been reduced after using alcohol for some of the students. To fully explore the causal relationship in this population, we need to assess the longitudinal association, which is one of the aims of our future studies.

Finally, it is worth mentioning that our sample had a high rate of females (70.4%), which may be related to two contributing factors: (1) the higher proportion of females in the population of undergraduates in this university (60%); and (2) the higher participation of females in this kind of studies (70). For instance, in a recent meta-analysis, the mean proportion of females (adjusted by the size of the study) was 66% (8). It is known that there are sex differences in the neurobiological mechanisms involved in stress, anxiety and affective disorders, where females have an increased risk of presenting these symptoms (71, 72). Furthermore, a study in the general population in Chile found that females were more likely to have a new mental health disorder during the pandemic than males (73).

This study has several strengths. First, it includes a large sample that invited to participate the whole body of students from the different academic units and enrollment years. Second, we had included several variables from different domains to explore the main risk and protective factors and reduce confounding bias. Finally, there are still few studies exploring the effect of pandemic and sanitary measures on mental health among undergraduate students in Latin American countries and in the world, making it an important contribution to research in the field (74). Among the limitations, we could mention that our data come from a cross-sectional survey, and no causality can be implied. Additionally, we used a self-reported questionnaire, which may introduce some reported bias. Moreover, not all the instruments used in this study were validated among Chilean undergraduates, and no diagnostic statements can be formulated because the instruments and methodology used are usually used to screen mental health issues. Our findings are based on data from one university and may therefore not be generalized to other universities or college students in Chile due to some cluster bias. In addition, our findings cannot be generalized to other phases of the pandemic, but only when lockdown measures were in place. Most students attending this university come from high-income families, which may also reduce the representativity of the results. Finally, the sample had a high rate of females, which may have increased the prevalence of mental health symptoms reported in the study. As anonymity was granted, it was impossible to directly contact high suicide risk students; however, a suggestion for seeking help and information was given at the end of the survey as we explained in Section Method.

Future research among university students can be concentrated on studies exploring the progression of mental health problems and the associated factors over time through longitudinal studies. Additionally, studies exploring the effectiveness of preventive interventions programs using some of the potential mediators found in this research, such as a sense of university belonging, should be conducted in the short term. The college environment offers an excellent opportunity to introduce interventions to prevent mental health problems, substance misuse, and bullying victimization. Additionally, all universities will need to be prepared to implement this kind of intervention when students return to the campus during and after the pandemic emergency.

## Data Availability Statement

The raw data supporting the conclusions of this article will be made available by the authors, without undue reservation.

## Ethics Statement

The studies involving human participants were reviewed and approved by Scientific Ethical Committee Universidad de los Andes. The patients/participants provided their written informed consent to participate in this study.

## Author Contributions

JV: writing—conceptualization, methodology, validation, formal analysis, investigation, data curation, review editing, and project administration and funding acquisition. FD, PC, GL, FS, MA, DN, and RA: writing—review and editing. SR: software, formal analysis, and writing—original draft. JG: conceptualization, methodology, validation, formal analysis, investigation, data curation, writing—review and editing, visualization, supervision, project administration, and funding acquisition. All authors contributed to the article and approved the submitted version.

## Funding

This research was funded by Fondo de Desarrollo Institucional, línea Emprendimiento Estudiantil 2019, UAN 1901. Ministry of Education, Chile; Social Responsibility Department, Universidad de los Andes, Chile, and by ANID—Millennium Science Initiative Program—NCS2021_081.

## Conflict of Interest

The authors declare that the research was conducted in the absence of any commercial or financial relationships that could be construed as a potential conflict of interest.

## Publisher's Note

All claims expressed in this article are solely those of the authors and do not necessarily represent those of their affiliated organizations, or those of the publisher, the editors and the reviewers. Any product that may be evaluated in this article, or claim that may be made by its manufacturer, is not guaranteed or endorsed by the publisher.

## Abbreviations

YLDs: years lived with disability
DASS-21: Depression, Anxiety, and Stress Scale
SARS-CoV-2: Severe Acute Respiratory Syndrome Coronavirus 2
ADHD: Attention Deficit Disorder with Hyperactivity
ASSIST, Alcohol: Smoking and Substance Involvement Screen Test
SD: Standard deviation
SSAF: Sense of Social and Academic Fit tool
ISI: Insomnia Severity Index
C-SSRS: Columbia-Suicide Severity Rating Scale.
