# Supplementary material for: Mental Health and Related Factors Among Undergraduate Students During SARS-CoV-2 Pandemic: A Cross-Sectional Study
Source: Front Psychiatry. 2022 May 31;13:833263. doi: 10.3389/fpsyt.2022.833263 (PMC9193581; doi:10.3389/fpsyt.2022.833263)
Supplement: Supplementary file 1 [file Table_1.DOCX]

Supplement Table 1: Univariable associations between risk and protective factors and the outcomes Depression, Anxiety, Stress, Insomnia, and Suicide risk.

| **Risk and protective factors** | Depression | Anxiety | Stress | Insomnia | Suicide risk |
| --- | --- | --- | --- | --- | --- |
|  | OR [95% CI]  p-value | OR [95% CI]  p-value | OR [95% CI]  p-value | OR [95% CI]  p-value | OR [95% CI]  p-value |
| **Personal domain** |  |  |  |  |  |
| Sex (ref=Male) |  |  |  |  |  |
| Female | 1.27 [1.10-1.46]  0.001 | 2.01 [1.74-2.32]  < 0.001 | 1.72 [1.50-1.96]  < 0.001 | 1.25 [1.10-1.44]  0.003 | 1.11 [0.94-1.31]  0.238 |
| Age | 0.97 [0.95-0.99]  0.030 | 0.96 [0.93-0.98]  0.001 | 0.98 [0.96-1.01]  0.205 | 0.97 [0.95-1.00]  0.064 | 1.02 [0.99-1.05]  0.237 |
| Nationality (ref=Chilean) |  |  |  |  |  |
| Other | 1.04 [0.73-1.47]  0.842 | 1.16 [0.83-1.64]  0.386 | 0.90 [0.65-1.27]  0.560 | 0.96 [0.67-1.38]  0.827 | 0.94 [0.61-1.44]  0.777 |
| Ethnicity (ref=Non-indigenous) |  |  |  |  |  |
| Indigenous | 1.47 [0.94-2.30]  0.088 | 1.49 [0.96-2.32]  0.077 | 1.19 [0.76-1.87]  0.448 | 1.18 [0.75-1.88]  0.471 | 1.31 [0.79-2.19]  0.301 |
| Occupational status (ref=Studying only) | * 0.3175 | *0.0057 | *0.0285 | *0.0265 | *0.0001 |
| Studying and part-time job | 1.16 [0.95-1.41]  0.141 | 1.33 [1.09-1.61]  0.004 | 1.30 [1.07-1.57]  0.009 | 1.27 [1.04-1.55]  0.020 | 1.53 [1.23-1.90]  < 0.001 |
| Studying and full-time job | 1.13 [0.63-2.03]  0.674 | 1.56 [0.88-2.77]  0.124 | 1.20 [0.68-2.14]  0.531 | 1.53 [0.86-2.73]  0.148 | 2.08 [1.14-3.81]  0.018 |
| History of chronic illness (ref=No) |  |  |  |  |  |
| Yes | 1.28 [1.10-1.50]  0.002 | 1.81 [1.55-2.11]  < 0.001 | 1.37 [1.18-1.61]  < 0.001 | 1.46 [1.25-1.71]  < 0.001 | 1.48 [1.24-1.77]  < 0.001 |
| History of mental health disorders |  |  |  |  |  |
| Depression | 3.04 [2.64-3.49]  < 0.001 | 2.58 [2.25-2.96]  < 0.001 | 2.44 [2.11-2.81]  < 0.001 | 1.89 [1.65-2.18]  < 0.001 | 3.48 [2.97-4.07]  < 0.001 |
| Bipolar disorder | 3.71 [2.56-5.39]  < 0.001 | 2.61 [1.82-3.74]  < 0.001 | 2.41 [1.62-3.58]  < 0.001 | 2.42 [1.70-3.45]  < 0.001 | 4.42 [3.10-6.31]  < 0.001 |
| Panic disorders | 2.48 [2.15-2.88]  < 0.001 | 4.18 [3.59-4.86]  < 0.001 | 2.90 [2.47-3.40]  < 0.001 | 2.16 [1.87-2.51]  < 0.001 | 2.26 [1.92-2.67]  < 0.001 |
| Anxiety disorders | 2.55 [2.24-2.90]  < 0.001 | 3.36 [2.94-3.83]  < 0.001 | 3.22 [2.81-3.69]  < 0.001 | 1.97 [1.73-2.26]  < 0.001 | 2.63 [2.26-3.07]  < 0.001 |
| Eating disorders | 2.40 [1.96-2.93]  < 0.001 | 2.42 [1.98-2.96]  < 0.001 | 2.32 [1.87-2.89]  < 0.001 | 1.91 [1.56-2.33]  < 0.001 | 2.09 [1.68-2.59]  < 0.001 |
| ADHD | 1.29 [1.12-1.47]  < 0.001 | 1.27 [1.11-1.45]  < 0.001 | 1.24 [1.09-1.42]  0.001 | 1.59 [1.39-1.82]  < 0.001 | 1.28 [1.09-1.50]  0.002 |
| History of mental health treatment |  |  |  |  |  |
| Psychotherapy | 1.89 [1.66-2.14]  < 0.001 | 1.90 [1.68-2.16]  < 0.001 | 1.92 [1.70-2.18]  < 0.001 | 1.43 [1.26-1.63]  < 0.001 | 2.16 [1.85-2.52]  < 0.001 |
| Pharmacologic | 2.01 [1.77-2.29]  < 0.001 | 1.94 [1.71-2.21]  < 0.001 | 1.97 [1.73-2.25]  < 0.001 | 1.73 [1.51-1.97]  < 0.001 | 2.09 [1.80-2.44]  < 0.001 |
| Physical activity during the week (ref=≤ 149 minutes |  |  |  |  |  |
| ≥ 150 minutes | 0.62 [0.55-0.71]  < 0.001 | 0.65 [0.57-0.74]  < 0.001 | 0.75 [0.66-0.85]  < 0.001 | 0.74 [0.65-0.85]  < 0.001 | 0.56 [0.48-0.66]  < 0.001 |
| Sexual orientation (ref=Heterosexual) |  |  |  |  |  |
| Homosexual | 2.45 [1.52-3.93]  < 0.001 | 1.97 [1.23-3.16]  0.005 | 1.87 [1.13-3.09]  0.014 | 1.17 [0.72-1.92]  0.523 | 4.71 [2.93-7.56]  < 0.001 |
| Bisexual | 2.77 [2.09-3.67]  < 0.001 | 2.60 [1.96-3.44]  < 0.001 | 2.05 [1.52-2.77]  < 0.001 | 1.72 [1.31-2.28]  < 0.001 | 4.33 [3.27-5.73]  < 0.001 |
| Unsure | 2.36 [1.71-3.25]  < 0.001 | 2.09 [1.51-2.88]  < 0.001 | 2.10 [1.48-3.0]  < 0.001 | 1.27 [0.91-1.76]  0.164 | 3.70 [2.67-5.13]  < 0.001 |
| Other | 1.90 [0.82-4.39]  0.134 | 2.62 [1.11-6.14]  0.027 | 1.29 [0.55-3.04]  0.553 | 1.23 [0.52-2.95]  0.636 | 4.84 [2.09-11.22]  < 0.001 |
| Offspring (ref=No children) |  |  |  |  |  |
| With children | 0.90 [0.49-1.67]  0.748 | 1.15 [0.64-2.08]  0.636 | 1.19 [0.66-2.14]  0.571 | 1.00 [0.54-1.87]  0.989 | 0.82 [0.38-1.77]  0.614 |
| Number of sex partners in the last year (ref=None) | *0.0109 | *0.0001 | *0.0001 | *0.1155 | *0.0002 |
| 1 or 2 | 1.16 [1.02-1.32]  0.028 | 1.32 [1.16-1.51]  < 0.001 | 1.38 [1.22-1.57]  < 0.001 | 1.13 [0.99-1.29]  0.075 | 1.15 [0.98-1.35]  0.080 |
| 3 or more | 1.48 [1.09-2.01]  0.011 | 1.32 [0.97-1.79]  0.080 | 1.46 [1.07-1.98]  0.016 | 1.27 [0.93-1.74]  0.139 | 2.01 [1.44-2.80]  < 0.001 |
| Substance use |  |  |  |  |  |
| Last month tobacco use | - 1. [0.86-1.17]   0.948 | 1.14 [0.98-1.33]  0.098 | 1.02 [0.88-1.19]  0.767 | 1.31 [1.12-1.53]  0.001 | 1.07 [0.89-1.29]  0.450 |
| Risk of tobacco use disorder | 1.64 [0.92-2.91]  0.093 | 2.92 [1.61-5.32]  < 0.001 | 2.75 [1.40-5.42]  0.003 | 2.01 [1.13-3.57]  0.018 | 2.04 [1.11-3.74]  0.022 |
| Last month alcohol use | 0.72 [0.64-0.82]  < 0.001 | 0.75 [0.66-0.85]  < 0.001 | 0.77 [0.68-0.87]  < 0.001 | 0.96 {0.84-1.09]  0.491 | 0.73 [0.63-0.85]  < 0.001 |
| Risk of alcohol use disorder | 1.15 [0.99-1.33]  0.067 | 1.11 [0.96-1.29]  0.157 | 1.09 [0.95-1.26]  0.222 | 1.28 [1.10-1.49]  0.001 | 1.46 [1.23-1.73]  < 0.001 |
| Last month cannabis use | 1.42 [1.13-1.77]  0.002 | 1.32 [1.06-1.65]  0.014 | 1.51 [1.20-1.90]  < 0.001 | 1.22 [0.97-1.53]  0.089 | 1.71 [1.33-2.18]  < 0.001 |
| Risk of cannabis use disorder | 2.49 [1.15-5.37]  0.020 | 2.06 [0.96-4.40]  0.064 | 2.93 [1.18-7.28]  0.020 | 1.94 [0.91-4.13]  0.087 | 3.16 [1.47-6.77]  0.003 |
| Last month tranquilizers use | 2.74 [2.20-3.42]  < 0.001 | 3.10 [2.48-3.87]  < 0.001 | 2.87 [2.24-3.68]  < 0.001 | 4.28 [3.42-5.37]  < 0.001 | 2.46 [1.95-3.09]  < 0.001 |
| Risk of tranquilizers use disorder | 7.91 [3.00-20.90]  < 0.001 | 7.62 [2.89-20.09]  < 0.001 | 22.74 [3.09-167.5]  0.002 | 9.70 [3.68-25.56]  < 0.001 | 4.57 [2.17-9.64]  < 0.001 |
| Last month nootropics use | 1.61 [1.30-1.99]  < 0.001 | 2.15 [1.73-2.66]  < 0.001 | 1.61 [1.29-2.01]  < 0.001 | 1.91 [1.54-2.37]  < 0.001 | 1.52 [1.20-1.93]  0.001 |
| Risk of nootropics use disorder | 4.20 [1.93-9.14]  < 0.001 | 3.00 [1.43-6.28]  0.004 | 7.86 [2.38-25.88]  0.001 | 2.54 [1.25-5.17]  0.010 | 2.16 [1.03-4.53]  0.041 |
| **Family domain** |  |  |  |  |  |
| Mother’s educational level (ref=Incomplete elementary school) | *0.0001 | *0.0095 | *0.0122 | *0.0934 | *0.0032 |
| Complete elementary school and incomplete high school | 0.85 [0.25-2.92]  0.794 | 0.66 [0.19-2.25]  0.503 | 0.25 [0.10-1.27]  0.095 | 1.52 [0.44-5.22]  0.503 | 0.50 [0.14-1.70]  0.265 |
| Complete high school and incomplete university | 0.38 [0.13-1.15]  0.088 | 0.37 [0.12-1.13]  0.080 | 0.23 [0.10-1.03]  0.054 | 0.95 [0.32-2.87]  0.934 | 0.42 [0.14-1.21]  0.107 |
| Complete university and incomplete postgraduate | 0.30 [0.10-0.90]  0.032 | 0.32 [0.11-0.97]  0.044 | 0.19 [0.04-0.86]  0.031 | 0.81 [0.27-2.43]  0.714 | 0.32 [0.11-0.92]  0.035 |
| Complete postgraduate | 0.31 [0.10-0.92]  0.035 | 0.31 [0.10-0.95]  0.040 | 0.18 [0.04-0.80]  0.025 | 0.88 [0.29-2.64]  0.813 | 0.30 [0.10-0.88]  0.029 |
| Father’s educational level (ref=Incomplete elementary school) | *0.0001 | *0.0001 | *0.0002 | *0.2321 | *0.0044 |
| Complete elementary school and incomplete high school | 1.29 [0.26-6.43]  0.755 | 1.18 [0.24-5.89]  0.836 | 1.11 [0.19-6.43]  0.909 | 0.87 [0.18-4.35]  0.869 | 1.37 [0.24-7.84]  0.723 |
| Complete high school and incomplete university | 0.51 [0.11-2.29]  0.379 | 0.55 [0.12-2.47]  0.435 | 0.59 [0.11-3.06]  0.529 | 0.72 [0.16-3.26]  0.675 | 0.76 [0.15-3.95]  0.745 |
| Complete university and incomplete postgraduate | 0.42 [0.10-1.88]  0.256 | 0.43 [0.10-1.92]  0.267 | 0.46 [0.10-2.40]  0.359 | 0.61 [0.14-2.74]  0.519 | 0.61 [0.12-3.17]  0.558 |
| Complete postgraduate | 0.39 [0.10-1.74]  0.216 | 0.41 [0.09-1.85]  0.246 | 0.42 [0.10-2.17]  0.299 | 0.62 [0.14-2.76]  0.527 | 0.56 [0.11-2.90]  0.488 |
| Family history of mental health problems |  |  |  |  |  |
| Psychiatric disorders | 1.53 [1.35-1.74]  < 0.001 | 1.71 [1.50-1.94]  < 0.001 | 1.60 [1.41-1.81]  < 0.001 | 1.35 [1.18-1.53]  < 0.001 | 2.04 [1.75-2.37]  < 0.001 |
| Suicide | 1.47 [1.22-1.78]  < 0.001 | 1.40 [1.15-1.69]  0.001 | 1.38 [1.14-1.68]  0.001 | 1.36 [1.12-1.66]  0.002 | 2.03 [1.65-2.50]  < 0.001 |
| Alcohol abuse or dependence | 1.54 [1.35-1.77]  < 0.001 | 1.63 [1.43-1.87]  < 0.001 | 1.49 [1.30-1.70]  < 0.001 | 1.36 [1.19-1.57]  < 0.001 | 2.01 [1.72-2.35]  < 0.001 |
| Drug abuse or dependence | 1.52 [1.26-1.84]  < 0.001 | 1.70 [1.40-2.05]  < 0.001 | 1.55 [1.27-1.88]  < 0.001 | 1.33 [1.09-1.61]  0.004 | 2.22 [1.81-2.72]  < 0.001 |
| Family functioning (APGAR) (ref=Highly functional) | *0.0001 | *0.0001 | *0.0001 | *0.0001 | *0.0001 |
| Moderate dysfunctional | 2.72 [2.31-3.19]  < 0.001 | 2.02 [1.72-2.37]  < 0.001 | 2.21 [1.87-2.61]  < 0.001 | 1.57 [1.33-1.85]  < 0.001 | 3.03 [2.52-3.65]  < 0.001 |
| Severe dysfunctional | 5.55 [4.48-6.87]  < 0.001 | 3.23 [2.64-3.96]  < 0.001 | 4.24 [3.34-5.38]  < 0.001 | 2.12 [1.73-2.60]  < 0.001 | 6.81 [5.50-8.44]  < 0.001 |
| **University domain** |  |  |  |  |  |
| Academic year (ref=1st) | *0.0004 | *0.0030 | *0.3710 | *0.1945 | *0.3511 |
| 2nd | 1.09 [0.90-1.32]  0.385 | 1.08 [0.89-1.30]  0.452 | 1.05 [0.87-1.26]  0.633 | 1.03 [0.84-1.25]  0.806 | 1.03 [0.82-1.30]  0.804 |
| 3rd | 0.98 [0.82-1.18]  0.851 | 0.99 [0.82-1.19]  0.873 | 0.98 [0.82-1.18]  0.847 | 1.08 [0.89-1.31]  0.439 | 1.16 [0.93-1.45]  0.184 |
| 4th | 0.79 [0.65-0.96]  0.015 | 0.90 [0.74-1.08]  0.260 | 0.91 [0.76-1.10 0.326 | 0.97 [0.79-1.18]  0.743 | 0.91 [0.72-1.15]  0.409 |
| 5th or higher | 0.74 [0.60-0.90]  0.002 | 0.73 [0.60-0.89]  0.002 | 0.87 [0.72-1.05]  0.155 | 0.84 [0.68-1.03]  0.091 | 1.00 [0.79-1.27]  0.991 |
| Source of financing |  |  |  |  |  |
| Funding from parents | 0.67 [0.55-0.82]  < 0.001 | 0.74 [0.61-0.90]  0.003 | 0.74 [0.61-0.91]  0.003 | 0.74 [0.60-0.90]  0.003 | 0.71 [0.57-0.90]  0.004 |
| Credit/Loan | 1.18 [1.03-1.34]  0.015 | 1.13 [0.99-1.29]  0.063 | 1.18 [1.03-1.34]  0.013 | 1.10 [0.96-1.26]  0.171 | 1.26 [1.10-1.47]  0.004 |
| Scholarship | 1.29 [1.13-1.47]  < 0.001 | 1.23 [1.07-1.40]  0.003 | 1.24 [1.09-1.41]  0.001 | 1.04 [0.90-1.19]  0.598 | 1.30 [1.11-1.52]  0.001 |
| Self-funded | 1.90 [1.43-2.51]  < 0.001 | 2.38 [1.79-3.16]  < 0.001 | 2.47 [1.80-3.39]  < 0.001 | 2.28 [1.71-3.02]  < 0.001 | 2.17 [1.61-2.92]  < 0.001 |
| Other means | 1.54 [1.15-2.07]  0.003 | 1.24 [0.93-1.67]  0.146 | 1.33 [0.99-1.80]  0.058 | 1.47 [1.09-1.98]  0.011 | 1.55 [1.12-2.14]  0.009 |
| Commuting time university (ref=±1SD) | *0.0001 | *0.0001 | *0.0001 | *0.0002 | *0.0001 |
| >1SD | 1.75 [1.49-2.05]  < 0.001 | 1.89 [1.61-2.21]  < 0.001 | 1.69 [1.44-2.00]  < 0.001 | 1.40 [1.19-1.65]  < 0.001 | 1.80 [1.50-2.16]  < 0.001 |
| < -1SD | 0.89 [0.73-1.08]  0.232 | 1.00 [0.83-1.21]  0.981 | 0.86 [0.72-1.03]  0.106 | 0.99 [0.82-1.21]  0.955 | 0.90 [0.71-1.14]  0.389 |
| History of failing subjects (ref=No failed subjects) |  |  |  |  |  |
| Failed subjects | 1.24 [1.09-1.41]  0.001 | 1.10 [0.97-1.26]  0.134 | 1.14 [1.00-1.29]  0.043 | 1.41 [1.23-1.61]  < 0.001 | 1.44 [1.24-1.68]  < 0.001 |
| Violence victimization |  |  |  |  |  |
| Physical | 4.00 [1.82-8.76]  0.001 | 6.63 [2.70-16.25]  < 0.001 | 7.57 [2.29-25.00]  0.001 | 4.20 [1.96-9.00]  < 0.001 | 5.97 [2.86-12.43]  < 0.001 |
| Psychological | 2.42 [1.97-2.98]  < 0.001 | 2.59 [2.11-3.19]  < 0.001 | 3.02 [2.38-3.82]  < 0.001 | 1.51 [1.23-1.86]  < 0.001 | 2.94 [2.37-3.64]  < 0.001 |
| Exclusion | 2.83 [2.47-3.25]  < 0.001 | 2.58 [2.26-2.96]  < 0.001 | 2.79 [2.42-3.21]  < 0.001 | 1.77 [1.54-2.03]  < 0.001 | 3.25 [2.79-3.80]  < 0.001 |
| Teasing | 2.29 [1.89-2.77]  < 0.001 | 2.26 [1.86-2.73]  < 0.001 | 2.30 [1.87-2.82]  < 0.001 | 1.82 [1.50-2.20]  < 0.001 | 2.45 [2.00-3.01]  < 0.001 |
| Ridiculization | 2.11 [1.74-2.55]  < 0.001 | 2.14 [1.77-2.59]  < 0.001 | 2.38 [1.93-2.92]  < 0.001 | 1.60 [1.32-1.93]  < 0.001 | 2.48 [2.03-3.04]  < 0.001 |
| Psychological sense of university belonging (ref = ±1SD) | *0.0001 | *0.0001 | *0.0001 | *0.0001 | *0.0001 |
| >1SD | 0.42 [0.34-0.52]  < 0.001 | 0.55 [0.45-0.67]  < 0.001 | 0.49 [0.41-0.59]  < 0.001 | 0.58 [0.48-0.71]  < 0.001 | 0.47 [0.35-0.62]  < 0.001 |
| < -1SD | 3.19 [2.69-3.79]  < 0.001 | 2.31 [1.96-2.74]  < 0.001 | 2.70 [2.23-3.25]  < 0.001 | 1.46 [1.23-1.73]  < 0.001 | 3.16 [2.64-3.78]  < 0.001 |
| **SARS-CoV-2 experiences domain** |  |  |  |  |  |
| History of personal contagion of SARS-CoV-2 (ref=No) |  |  |  |  |  |
| Yes | 1.07 [0.75-1.53]  0.703 | 1.03 [0.72-1.47]  0.865 | 0.97 [0.68-1.37]  0.857 | 0.81 [0.55-1.19]  0.290 | 0.83 [0.52-1.31]  0.417 |
| History of family contagion of SARS-CoV-2 (ref=No) |  |  |  |  |  |
| Yes | 1.14 [0.99-1.30]  0.067 | 1.22 [1.07-1.40]  0.003 | 1.26 [1.10-1.44]  0.001 | 1.19 [1.04-1.37]  0.015 | 1.26 [1.07-1.48]  0.005 |
| Fear to contracting SARS-CoV-2 by the students (ref=Not at all) | *0.0001 | *0.0001 | *0.0001 | *0.0001 | *0.0001 |
| Slightly | 0.86 [0.66-1.11]  0.249 | 0.90 [0.68-1.19]  0.457 | 1.02 [0.79-1.32]  0.856 | 0.98 [0.74-1.30]  0.896 | 0.58 [0.43-0.78]  < 0.001 |
| Somewhat | 0.76 [0.59-0.97]  0.029 | 1.14 [0.88-1.47]  0.332 | 1.25 [0.99-1.58]  0.066 | 1.02 [0.78-1.33]  0.887 | 0.52 [0.40-0.69]  < 0.001 |
| Moderately | 1.15 [0.89-1.47]  0.279 | 1.74 [1.34-2.26]  < 0.001 | 1.91 [1.49-2.44]  < 0.001 | 1.37 [1.04-1.79]  0.024 | 0.68 [0.52-0.91]  0.009 |
| Extremely | 1.97 [1.50-2.61]  < 0.001 | 3.41 [2.55-4.54]  < 0.001 | 3.38 [2.54-4.50]  < 0.001 | 2.71 [2.02-3.63]  < 0.001 | 1.10 [0.81-1.49]  0.546 |
| Fear of others contracting SARS-CoV-2 (ref=Not at all) | *0.0001 | *0.0001 | *0.0001 | *0.0001 | *0.0001 |
| Slightly | 0.34 [0.17-0.68]  0.002 | 0.61 [0.28-1.33]  0.213 | 0.89 [0.44-1.79]  0.749 | 0.65 [0.31-1.37]  0.259 | 0.52 [0.24-1.11]  0.090 |
| Somewhat | 0.34 [0.18-0.62]  0.001 | 0.93 [0.47-1.81]  0.825 | 1.22 [0.66-2.27]  0.521 | 0.58 [0.30-1.10]  0.096 | 0.40 [0.21-0.77]  0.006 |
| Moderately | 0.40 [0.22-0.72]  0.002 | 1.06 [0.55-2.03]  0.868 | 1.45 [0.79-2.65]  0.231 | 0.75 [0.40-1.42]  0.379 | 0.39 [0.21-0.74]  0.004 |
| Extremely | 0.69 [0.38-1.25]  0.224 | 2.28 [1.19-4.37]  0.013 | 2.53 [1.38-4.62]  0.003 | 1.32 [0.70-2.47]  0.387 | 0.63 [0.34-1.19]  0.154 |
| Living condition during pandemic lockdowns (ref=Living with family, friends or roommates) |  |  |  |  |  |
| Living independently | 1.31 [0.83-2.07]  0.248 | 1.41 [0.89-2.22]  0.143 | 1.15 [0.73-1.82]  0.556 | 1.44 [0.91-2.29]  0.122 | 1.22 [0.71-2.08]  0.471 |
| Frequency of social contact during lockdowns (ref=Never) | *0.0001 | *0.0004 | *0.0001 | *0.0459 | *0.0001 |
| 1-2 days a week | 0.60 [0.45-0.79]  < 0.001 | 0.58 [0.43-0.76]  < 0.001 | 0.64 [0.48-0.86]  0.003 | 0.98 [0.73-1.32]  0.907 | 0.63 [0.46-0.86]  0.004 |
| 3-4 days a week | 0.43 [0.32-0.59]  < 0.001 | 0.53 [0.39-0.72]  < 0.001 | 0.49 [0.36-0.67]  < 0.001 | 0.82 [0.60-1.13]  0.231 | 0.43 [0.30-0.61]  < 0.001 |
| 5-6 days a week | 0.50 [0.35-0.71]  < 0.001 | 0.46 [0.32-0.66]  < 0.001 | 0.57 [0.40-0.82]  0.002 | 0.73 [0.50-1.06]  0.095 | 0.45 [0.30-0.68]  < 0.001 |
| Everyday | 0.50 [0.37-0.69]  < 0.001 | 0.60 [0.43-0.82]  0.001 | 0.56 [0.41-0.78]  0.001 | 1.04 [0.74-1.45]  0.835 | 0.64 [0.45-0.91]  0.013 |
| Frequency of physical exercising during lockdowns (ref=Never) | *0.0001 | *0.0001 | *0.0001 | *0.0001 | *0.0001 |
| 1-2 days a week | 0.69 [0.58-0.83]  < 0.001 | 0.78 [0.66-0.93]  0.006 | 0.77 [0.65-0.92]  0.004 | 0.93 [0.78-1.11]  0.409 | 0.79 [0.65-0.97]  0.022 |
| 3-4 days a week | 0.54 [0.45-0.64]  < 0.001 | 0.66 [0.56-0.79]  < 0.001 | 0.70 [0.59-0.83]  < 0.001 | 0.64 [0.54-0.77]  < 0.001 | 0.55 [0.45-0.68]  < 0.001 |
| 5-6 days a week | 0.44 [0.36-0.53]  < 0.001 | 0.53 [0.43-0.65]  < 0.001 | 0.57 [0.47-0.69]  < 0.001 | 0.61 [0.49-0.75]  < 0.001 | 0.45 [0.35-0.58]  < 0.001 |
| Everyday | 0.39 [0.26-0.58]  < 0.001 | 0.46 [0.31-0.68]  < 0.001 | 0.55 [0.38-0.79]  0.001 | 0.44 [0.29-0.67]  < 0.001 | 0.42 [0.25-0.69]  0.001 |
| Frequency of recreational activities during lockdowns (ref=Never) | *0.0001 | *0.0001 | *0.0001 | *0.0001 | *0.3672 |
| 1-2 days a week | 0.62 [0.53-0.73]  < 0.001 | 0.77 [0.66-0.90]  0.001 | 0.78 [0.66-0.91]  0.002 | 0.79 [0.67-0.93]  0.004 | 0.91 [0.75-1.10]  0.340 |
| 3-4 days a week | 0.51 [0.43-0.62]  < 0.001 | 0.66 [0.55-0.80]  < 0.001 | 0.62 [0.51-0.74]  < 0.001 | 0.56 [0.46-0.68]  < 0.001 | 0.83 [0.66-1.04]  0.100 |
| 5-6 days a week | 0.40 [0.31-0.53]  < 0.001 | 0.67 [0.52-0.87]  0.003 | 0.57 [0.44-0.73]  < 0.001 | 0.56 [0.43-0.74]  < 0.001 | 0.83 [0.61-1.13]  0.241 |
| Everyday | 0.39 [0.30-0.51]  < 0.001 | 0.53 [0.40-0.68]  < 0.001 | 0.45 [0.35-0.57]  < 0.001 | 0.55 [0.42-0.73]  < 0.001 | 0.78 [0.57-1.06]  0.117 |
| Keeping a routine during lockdowns (ref=Never) | *0.0001 | *0.0001 | *0.0001 | *0.0001 | *0.0001 |
| 1-2 days a week | 0.70 [0.56-0.88]  0.002 | 0.67 [0.53-0.85]  0.001 | 0.66 [0.52-0.83]  0.001 | 0.64 [0.51-0.81]  < 0.001 | 0.75 [0.58-0.97]  0.029 |
| 3-4 days a week | 0.45 [0.37-0.54]  < 0.001 | 0.61 [0.50-0.74]  < 0.001 | 0.61 [0.50-0.74]  < 0.001 | 0.51 [0.42-0.62]  < 0.001 | 0.54 [0.43-0.68]  < 0.001 |
| 5-6 days a week | 0.33 [0.27-0.40]  < 0.001 | 0.46 [0.38-0.56]  < 0.001 | 0.45 [0.37-0.55]  < 0.001 | 0.36 [0.29-0.44]  < 0.001 | 0.41 [0.32-0.52]  < 0.001 |
| Everyday | 0.35 [0.29-0.43]  < 0.001 | 0.48 [0.39-0.58]  < 0.001 | 0.47 [0.38-0.57]  < 0.001 | 0.37 [0.30-0.45]  < 0.001 | 0.46 [0.37-0.58]  < 0.001 |
| Frequency of meditation or praying during lockdowns (ref=Never) | *0.0001 | *0.0116 | *0.0022 | *0.0003 | *0.0014 |
| 1-2 days a week | 0.72 [0.62-0.84]  < 0.001 | 0.87 [0.75-1.01]  0.063 | 0.86 [0.74-0.99]  0.038 | 0.85 [0.73-0.99]  0.045 | 0.83 [0.69-0.99]  0.041 |
| 3-4 days a week | 0.84 [0.67-1.05]  0.133 | 1.19 [0.96-1.49]  0.112 | 1.21 [0.97-1.51]  0.091 | 0.85 [0.67-1.07]  0.165 | 1.10 [0.85-1.43]  0.447 |
| 5-6 days a week | 0.62 [0.44-0.87]  0.005 | 0.95 [0.69-1.30]  0.731 | 0.66 [0.49-0.90]  0.009 | 0.85 [0.61-1.19]  0.353 | 0.65 [0.43-0.99]  0.045 |
| Everyday | 0.50 [0.39-0.65]  < 0.001 | 0.74 [0.59-0.95]  0.016 | 0.83 [0.67-1.04]  0.115 | 0.55 [0.43-0.72]  < 0.001 | 0.60 [0.43-0.82]  0.001 |

Note: * = Walt test; n = number of participants; CI = Confidence Interval.

Supplement Table 2: Multivariable associations by domain between risk and protective factors and the outcomes Depression, Anxiety, Stress, Insomnia, and Suicide risk.

| **Risk and protective factors** | Depression | Anxiety | Stress | Insomnia | Suicide risk |
| --- | --- | --- | --- | --- | --- |
|  | OR [95% CI]  p-value | OR [95% CI]  p-value | OR [95% CI]  p-value | OR [95% CI]  p-value | OR [95% CI]  p-value |
| **Personal domain** |  |  |  |  |  |
| Sex (ref=Male) |  |  |  |  |  |
| Female | 1.00 [0.85-1.17]  0.958 | 1.55 [1.31-1.83]  < 0.001 | 1.41 [1.21-1.64]  < 0.001 | 1.06 [0.90-1.25]  0.464 | 0.94 [0.77-1.14]  0.526 |
| Age | 0.94 [0.91-0.87]  < 0.001 | 0.91 [0.88-0.94]  < 0.001 | 0.95 [0.92-0.98]  0.001 | 0.95 [0.92-0.98]  0.001 | 0.97 [0.93-1.00]  0.063 |
| Nationality (ref=Chilean) |  |  |  |  |  |
| Other | - | - | - | - | - |
| Ethnicity (ref=Non-indigenous) |  |  |  |  |  |
| Indigenous | - | - | - | - | - |
| Occupational status (ref=Studying only) |  | *0.0311 | *0.2126 | *0.0656 | *0.0054 |
| Studying and part-time job | - | 1.30 [1.04-1.62]  0.022 | 1.21 [0.97-1.50]  0.084 | 1.23 [0.99-1.53]  0.058 | 1.45 [1.13-1.86]  0.004 |
| Studying and full-time job | - | 1.71 [0.85-3.47]  0.134 | 1.18 [0.60-2.29]  0.634 | 1.66 [0.87-3.16]  0.123 | 1.83 [0.90-3.74]  0.094 |
| History of chronic illness (ref=No) |  |  |  |  |  |
| Yes | 1.06 [0.89-1.25]  0.527 | 1.48 [1.25-1.76]  < 0.001 | 1.12 [0.95-1.33]  0.184 | 1.28 [1.08-1.51]  0.005 | 1.24 [1.02-1.52]  0.032 |
| History of mental health disorders |  |  |  |  |  |
| Depression | 1.91 [1.61-2.28]  < 0.001 | 1.32 [1.10-1.58]  0.003 | 1.29 [1.08-1.55]  0.006 | 1.19 [0.99-1.42]  0.062 | 2.17 [1.77-2.66]  < 0.001 |
| Bipolar disorder | 1.55 [1.01-2.36]  0.043 | 1.03 [0.67-1.57]  0.896 | 1.01 [0.64-1.58]  0.962 | 1.25 [0.84-1.87]  0.277 | 1.76 [1.17-2.64]  0.007 |
| Panic disorders | 1.39 [1.16-1.66]  < 0.001 | 2.40 [2.00-2.88]  < 0.001 | 1.51 [1.25-1.83]  < 0.001 | 1.44 [1.20-1.72]  < 0.001 | 1.09 [0.89-1.35]  0.401 |
| Anxiety disorders | 1.49 [1.26-1.76]  < 0.001 | 1.96 [1.66-2.32]  < 0.001 | 2.10 [1.77-2.48]  < 0.001 | 1.30 [1.10-1.54]  0.003 | 1.53 [1.25-1.86]  0.000 |
| Eating disorders | 1.46 [1.15-1.81]  0.001 | 1.27 [1.00-1.60]  0.046 | 1.31 [1.02-1.66]  0.031 | 1.27 [1.02-1.59]  0.036 | 1.16 [0.90-1.48]  0.260 |
| ADHD | 1.07 [0.91-1.24]  0.427 | 1.01 [0.86-1.19]  0.875 | 1.08 [0.93-1.26]  0.331 | 1.33 [1.14-1.55]  < 0.001 | 0.99 [0.82-1.19]  0.917 |
| History of mental health treatment |  |  |  |  |  |
| Psychotherapy | 1.11 [0.93-1.33]  0.259 | 1.13 [0.94-1.36]  0.181 | 1.19 [1.00-1.41]  0.046 | 0.90 [0.75-1.09]  0.285 | 1.29 [1.03-1.60]  0.026 |
| Pharmacologic | 0.95 [0.78-1.15]  0.576 | 0.77 [0.62-0.94]  0.010 | 0.88 [0.73-1.07]  0.202 | 1.07 [0.88-1.31]  0.491 | 0.88 [0.70-1.12]  0.308 |
| Physical activity during the week (ref=≤ 149 minutes |  |  |  |  |  |
| ≥ 150 minutes | 0.67 [0.59-0.77]  < 0.001 | 0.69 [0.60-0.80]  < 0.001 | 0.80 [0.70-0.92]  0.001 | 0.80 [0.70-0.92]  0.002 | 0.63 [0.53-0.75]  < 0.001 |
| Sexual orientation (ref=Heterosexual) |  |  |  |  |  |
| Homosexual | 1.95 [1.16-3.27]  0.012 | 1.97 [1.15-3.36]  0.013 | 1.69 [0.98-2.92]  0.058 | 0.84 [0.49-1.45]  0.533 | 3.65 [2.15-6.19]  < 0.001 |
| Bisexual | 2.12 [1.57-2.86]  < 0.001 | 2.07 [1.52-2.82]  < 0.001 | 1.57 [1.15-2.16]  0.005 | 1.40 [1.05-1.88]  0.024 | 3.38 [2.49-4.58]  < 0.001 |
| Unsure | 2.04 [1.45-2.87]  < 0.001 | 1.86 [1.31-2.64]  0.001 | 1.83 [1.27-2.65]  0.001 | 1.18 [0.83-1.67]  0.351 | 3.31 [2.33-4.70]  < 0.001 |
| Other | 1.14 [0.46-2.81]  0.774 | 1.44 [0.54-3.81]  0.463 | 0.78 [0.31-1.98]  0.601 | 0.76 [0.30-1.94]  0.566 | 3.12 [1.26-7.72]  0.014 |
| Offspring (ref=No children) |  |  |  |  |  |
| With children | - | - | - | - | - |
| Number of sex partners in the last year (ref=None) | *0.2764 | *0.0037 | *0.0005 |  | *0.5192 |
| 1 or 2 | 1.12 [0.97-1.30]  0.119 | 1.28 [1.10-1.49]  0.001 | 1.32 [1.15-1.52]  < 0.001 | - | 1.06 [0.89-1.27]  0.499 |
| 3 or more | 1.15 [0.81-1.63]  0.425 | 1.02 [0.71-1.46]  0.926 | 1.14 [0.81-1.61]  0.439 | - | 1.24 [0.84-1.83]  0.277 |
| Substance use |  |  |  |  |  |
| Last month tobacco use | - | - | - | 1.07 [0.90-1.28]  0.424 | - |
| Risk of tobacco use disorder | - | 2.41 [1.22-4.75]  0.011 | 2.11 [1.01-4.40]  0.047 | 1.21 [0.64-2.31]  0.558 | 1.28 [0.63-2.58]  0.496 |
| Last month alcohol use | 0.72 [0.63-0.83]  < 0.001 | 0.77 [0.67-0.89]  < 0.001 | 0.77 [0.67-0.88]  < 0.001 | - | 0.64 [0.54-0.76]  < 0.001 |
| Risk of alcohol use disorder | - | - | - | 1.27 [1.08-1.50]  0.003 | 1.69 [1.39-2.06]  < 0.001 |
| Last month cannabis use | 1.37 [1.06-1.78]  0.017 | 1.36 [1.04-1.77]  0.023 | 1.44 [1.11-1.88]  0.007 | - | 1.39 [1.03-1.87]  0.030 |
| Risk of cannabis use disorder | 1.23 [0.51-2.96]  0.650 | - | 1.61 [0.58-4.45]  0.357 | - | 1.02 [0.39-2.61]  0.975 |
| Last month tranquilizers use | 1.56 [1.21-2.01]  0.001 | 1.63 [1.25-2.11]  < 0.001 | 1.61 [1.22-2.13]  0.001 | 3.02 [2.36-3.88]  < 0.001 | 1.32 [1.01-1.74]  0.045 |
| Risk of tranquilizers use disorder | 1.88 [0.65-5.46]  0.247 | 2.17 [0.73-6.44]  0.163 | 5.34 [0.68-41.89]  0.111 | 2.36 [0.82-6.79]  0.111 | 1.42 [0.57-3.52]  0.450 |
| Last month nootropics use | 1.15 [0.89-1.49]  0.288 | 1.81 [1.38-2.36]  < 0.001 | 1.10 [0.84-1.43]  0.483 | 1.19 [0.92-1.54]  0.190 | 1.13 [0.83-1.53]  0.431 |
| Risk of nootropics use disorder | 2.08 [0.82-5.28]  0.124 | 0.93 [0.37-2.37]  0.884 | 4.22 [1.15-15.55]  0.030 | 0.78 [0.32-1.90]  0.591 | 0.95 [0.37-2.43]  0.908 |
| **Family domain** |  |  |  |  |  |
| Sex (ref=Male) |  |  |  |  |  |
| Female | 1.26 [1.09-1.47]  0.002 | 2.01 [1.72-2.34]  < 0.001 | 1.74 [1.51-2.01]  < 0.001 | 1.22 [1.05-1.41]  0.009 | 1.05 [0.87-1.26]  0.626 |
| Age | 0.94 [0.92-0.97]  < 0.001 | 0.94 [0.91-0.96]  < 0.001 | 0.97 [0.94-1.00]  0.020 | 0.96 [0.94-0.99]  0.008 | 0.98 [0.95-1.02]  0.348 |
| Mother’s educational level (ref=Incomplete elementary school) | *0.0992 | *0.9676 | *0.5956 |  | *0.2929 |
| Complete elementary school and incomplete high school | 1.18 [0.26-5.29]  0.831 | 0.75 [0.17-3.36]  0.708 | 0.20 [0.02-1.83]  0.154 | - | 0.77 [0.17-3.52]  0.736 |
| Complete high school and incomplete university | 0.67 [0.17-2.66]  0.572 | 0.65 [0.17-2.57]  0.541 | 0.21 [0.03-1.78]  0.153 | - | 0.71 [0.18-2.79]  0.620 |
| Complete university and incomplete postgraduate | 0.57 [0.14-2.25]  0.421 | 0.65 [0.16-2.55]  0.534 | 0.20 [0.02-1.69]  0.140 | - | 0.57 [0.15-2.27]  0.428 |
| Complete postgraduate | 0.61 [0.15-2.43]  0.482 | 0.64 [0.16-2.56]  0.530 | 0.20 [0.02-1.64]  0.133 | - | 0.57 [0.14-2.30]  0.432 |
| Father’s educational level (ref=Incomplete elementary school) | *0.0896 | *0.0128 | *0.0318 | - | *0.6188 |
| Complete elementary school and incomplete high school | 1.59 [0.27-9.29]  0.604 | 1.29 [0.22-7.36]  0.778 | 1.54 [0.23-10.36]  0.658 | - | 1.69 [0.26-11.24]  0.585 |
| Complete high school and incomplete university | 0.83 [0.15-4.41]  0.823 | 0.66 [0.13-3.49]  0.629 | 0.93 [0.15-5.67]  0.938 | - | 1.22 [0.20-7.49]  0.827 |
| Complete university and incomplete postgraduate | 0.73 [0.14-3.91]  0.715 | 0.53 [0.10-2.81]  0.460 | 0.76 [0.12-4.64]  0.767 | - | 1.10 [0.18-6.73]  0.920 |
| Complete postgraduate | 0.70 [0.13-3.73]  0.672 | 0.53 [0.10-2.77]  0.448 | 0.71 [0.12-4.36]  0.714 | - | 1.07 [0.17-6.60]  0.940 |
| Family history of mental health problems |  |  |  |  |  |
| Psychiatric disorders | 1.25 [1.08-1.45]  0.003 | 1.46 [1.26-1.68]  < 0.001 | 1.33 [1.16-1.54]  < 0.001 | 1.16 [1.01-1.34]  0.038 | 1.44 [1.21-1.72]  < 0.001 |
| Suicide | 1.22 [0.98-1.51]  0.072 | 1.05 [0.85-1.30]  0.644 | 1.07 [0.87-1.33]  0.509 | 1.17 [0.95-1.44]  0.133 | 1.46 [1.15-1.85]  0.002 |
| Alcohol abuse or dependence | 1.19 [1.02-1.40]  0.032 | 1.24 [1.06-1.46]  0.008 | 1.15 [0.98-1.34]  0.092 | 1.18 [1.00-1.38]  0.043 | 1.36 [1.12-1.64]  0.002 |
| Drug abuse or dependence | 1.03 [0.82-1.29]  0.792 | 1.19 [0.95-1.48]  0.132 | 1.14 [0.91-1.43]  0.258 | 1.05 [0.85-1.31]  0.650 | 1.35 [1.05-1.73]  0.018 |
| Family functioning (APGAR) (ref=Highly functional) | *0.0001 | *0.0001 | *0.0001 | *0.0001 | *0.0001 |
| Moderate dysfunctional | 2.59 [2.19-3.06]  < 0.001 | 1.95 [1.64-2.31]  < 0.001 | 2.14 [1.80-2.55]  < 0.001 | 1.54 [1.30-1.82]  < 0.001 | 2.70 [2.23-3.28]  < 0.001 |
| Severe dysfunctional | 5.19 [4.16-6.48]  < 0.001 | 2.99 [2.41-3.70]  < 0.001 | 3.90 [3.05-4.99]  < 0.001 | 2.00 [1.63-2.46]  < 0.001 | 5.91 [4.73-7.39]  < 0.001 |
| **University domain** |  |  |  |  |  |
| Sex (ref=Male) |  |  |  |  |  |
| Female | 1.37 [1.18-1.60]  < 0.001 | 2.12 [1.82-2.48]  < 0.001 | 1.87 [1.62-2.17]  < 0.001 | 1.34 [1.15-1.56]  < 0.001 | 1.19 [0.99-1.43]  0.062 |
| Age | 0.99 [0.95-1.03]  0.508 | 0.94 [0.90-0.98]  0.003 | 0.94 [0.92-0.97]  < 0.001 | 0.92 [0.90-0.95]  < 0.001 | 0.97 [0.93-1.00]  0.068 |
| Academic year (ref=1st) | *0.0001 | *0.0496 |  |  |  |
| 2nd | 1.07 [0.87-1.33]  0.513 | 1.15 [0.94-1.42]  0.180 | - | - | - |
| 3rd | 0.82 [0.66-1.03]  0.082 | 0.97 [0.78-1.20]  0.774 | - | - | - |
| 4th | 0.65 [0.51-0.83]  0.001 | 0.94 [0.73-1.19]  0.585 | - | - | - |
| 5th or higher | 0.56 [0.42-0.75]  < 0.001 | 0.76 [0.57-1.01]  0.061 | - | - | - |
| Source of financing |  |  |  |  |  |
| Funding from parents | 0.95 [0.75-1.21]  0.669 | 1.02 [0.81-1.30]  0.849 | 1.02 [0.80-1.29]  0.883 | 0.88 [0.70-1.09]  0.240 | 1.05 [0.80-1.38]  0.721 |
| Credit/Loan | 0.99 [0.86-1.14]  0.895 | - | 1.00 [0.87-1.15]  0.965 | - | 1.01 [0.85-1.20]  0.916 |
| Scholarship | 1.14 [0.97-1.34]  0.102 | 1.02 [0.87-1.19]  0.816 | 1.09 [0.93-1.27]  0.268 | - | 1.16 [0.96-1.40]  0.114 |
| Self-funded | 1.42 [1.02-1.96]  0.035 | 2.23 [1.61-3.09]  < 0.001 | 2.28 [1.60-3.26]  < 0.001 | 2.18 [1.60-2.96]  < 0.001 | 1.59 [1.13-2.24]  0.008 |
| Other means | 1.20 [0.87-1.32]  0.268 | - | - | 1.20 [0.88-1.65]  0.255 | 1.18 [0.82-1.69]  0.366 |
| Commuting time university (ref=±1SD) | *0.0249 | *0.0001 | *0.0290 | *0.1943 | *0.0114 |
| >1SD | 1.28 [1.07-1.53]  0.007 | 1.50 [1.25-1.79]  < 0.001 | 1.28 [1.07-1.54]  0.008 | 1.16 [0.97-1.38]  0.096 | 1.36 [1.11-1.66]  0.003 |
| < -1SD | 1.07 [0.88-1.32]  0.496 | 1.18 [0.97-1.44]  0.106 | 1.01 [0.84-1.23]  0.896 | 1.10 [0.90-1.35]  0.329 | 1.13 [0.88-1.45]  0.346 |
| History of failing subjects (ref=No failed subjects) |  |  |  |  |  |
| Failed subjects | 1.27 [1.09-1.47]  0.002 | - | 1.16 [1.01-1.34]  0.038 | 1.48 [1.28-1.71]  < 0.001 | 1.28 [1.08-1.52]  0.005 |
| Violence victimization |  |  |  |  |  |
| Physical | 1.86 [0.79-4.37]  0.157 | 3.10 [1.19-8.06]  0.020 | 3.01 [0.87-10.43]  0.083 | 2.70 [1.22-5.98]  0.015 | 2.50 [1.11-5.60]  0.026 |
| Psychological | 1.47 [1.14-1.88]  0.003 | 1.63 [1.27-2.08]  < 0.001 | 1.77 [1.35-2.32]  < 0.001 | 1.01 [0.79-1.29]  0.924 | 1.55 [1.19-2.00]  0.001 |
| Exclusion | 1.86 [1.59-2.18]  < 0.001 | 1.81 [1.55-2.12]  < 0.001 | 1.84 [1.56-2.16]  < 0.001 | 1.40 [1.19-1.64]  < 0.001 | 2.00 [1.67-2.39]  < 0.001 |
| Teasing | 1.29 [1.01-1.66]  0.044 | 1.40 [1.09-1.80]  0.008 | 1.19 [0.92-1.55]  0.185 | 1.38 [1.08-1.76]  0.010 | 1.11 [0.84-1.45]  0.468 |
| Ridiculization | 1.19 [0.93-1.52]  0.177 | 1.20 [0.93-1.53]  0.159 | 1.29 [0.99-1.67]  0.056 | 1.08 [0.85-1.38]  0.525 | 1.29 [0.99-1.69]  0.061 |
| Psychological sense of university belonging (ref = ±1SD) | *0.0001 | *0.0001 | *0.0001 | *0.0001 | *0.0001 |
| >1SD | 0.48 [0.39-0.60]  < 0.001 | 0.63 [0.51-0.77]  < 0.001 | 0.56 [0.47-0.67]  < 0.001 | 0.65 [0.53-0.80]  < 0.001 | 0.56 [0.42-0.75]  < 0.001 |
| < -1SD | 2.32 [1.92-2.79]  < 0.001 | 1.73 [1.44-2.09]  < 0.001 | 1.98 [1.62-2.43]  < 0.001 | 1.13 [0.94-1.36]  0.187 | 2.13 [1.75-2.59]  < 0.001 |
| **SARS-CoV-2 experiences domain** |  |  |  |  |  |
| Sex (ref=Male) |  |  |  |  |  |
| Female | 1.22 [1.05-1.42]  0.010 | 1.85 [1.58-2.16]  < 0.001 | 1.58 [1.37-1.82]  < 0.001 | 1.14 [0.97-1.33]  0.104 | 1.09 [0.92-1.31]  0.317 |
| Age | 0.98 [0.95-1.01]  0.175 | 0.98 [0.95-1.00]  0.106 | 1.00 [0.98-1.03]  0.883 | 0.99 [0.97-1.02]  0.642 | 1.03 [1.00-1.06]  0.098 |
| History of personal contagion of SARS-CoV-2 (ref=No) |  |  |  |  |  |
| Yes | - | - | - | - | - |
| History of family contagion of SARS-CoV-2 (ref=No) |  |  |  |  |  |
| Yes | - | 1.18 [1.02-1.37]  0.022 | 1.24 [1.08-1.43]  0.003 | 1.16 [1.00-1.35]  0.043 | 1.22 [1.03-1.44]  0.019 |
| Fear to contracting SARS-CoV-2 by the students (ref=Not at all) | *0.0001 | *0.0001 | *0.0001 | *0.0001 | *0.0001 |
| Slightly | 0.95 [0.71-1.27]  0.722 | 0.89 [0.66-1.20]  0.459 | 1.01 [0.77-1.32]  0.970 | 1.03 [0.76-1.40]  0.839 | 0.63 [0.46-0.86]  0.004 |
| Somewhat | 0.75 [0.57-0.99]  0.045 | 1.00 [0.75-1.32]  0.976 | 1.13 [0.87-1.48]  0.347 | 0.97 [0.73-1.31]  0.856 | 0.54 [0.40-0.73]  < 0.001 |
| Moderately | 0.94 [0.70-1.26]  0.677 | 1.22 [0.91-1.65]  0.185 | 1.48 [1.12-1.96]  0.006 | 1.09 [0.80-1.48]  0.602 | 0.59 [0.42-0.82]  0.001 |
| Extremely | 1.37 [0.99-1.89]  0.057 | 2.03 [1.46-2.82]  < 0.001 | 2.27 [1.64-3.13]  < 0.001 | 1.89 [1.35-2.64]  < 0.001 | 0.81 [0.57-1.15]  0.239 |
| Fear of others contracting SARS-CoV-2 (ref=Not at all) | *0.0001 | *0.0001 | *0.0010 | *0.0001 | *0.0005 |
| Slightly | 0.39 [0.19-0.81]  0.012 | 0.76 [0.34-1.70]  0.505 | 1.09 [0.53-2.26]  0.810 | 0.66 [0.30-1.41]  0.281 | 0.69 [0.32-1.52]  0.358 |
| Somewhat | 0.38 [0.20-0.73]  0.004 | 1.07 [0.53-2.17]  0.855 | 1.37 [0.71-2.63]  0.348 | 0.55 [0.28-1.09]  0.086 | 0.55 [0.28-1.11]  0.093 |
| Moderately | 0.48 [0.25-0.91]  0.025 | 1.12 [0.56-2.27]  0.743 | 1.45 [0.76-2.78]  0.262 | 0.74 [0.38-1.45]  0.383 | 0.63 [0.32-1.24]  0.181 |
| Extremely | 0.68 [0.35-1.29]  0.236 | 1.79 [0.89-3.61]  0.103 | 1.90 [0.99-3.64]  0.055 | 1.04 [0.53-2.04]  0.920 | 0.91 [0.46-1.80]  0.781 |
| Living condition during pandemic lockdowns (ref=Living with family, friends or roommates) |  |  |  |  |  |
| Living independently | - | - | - | - | - |
| Frequency of social contact during lockdowns (ref=Never) | *0.0014 | *0.0030 | *0.0080 | *0.0894 | *0.0011 |
| 1-2 days a week | 0.68 [0.50-0.92]  0.012 | 0.59 [0.43-0.79]  0.001 | 0.66 [0.48-0.90]  0.009 | 1.11 [0.81-1.51]  0.531 | 0.73 [0.53-1.00]  0.052 |
| 3-4 days a week | 0.53 [0.39-0.74]  < 0.001 | 0.60 [0.43-0.83]  0.002 | 0.55 [0.39-0.76]  < 0.001 | 0.99 [0.70-1.39]  0.942 | 0.52 [0.36-0.75]  < 0.001 |
| 5-6 days a week | 0.57 [0.39-0.83]  0.003 | 0.47 [0.32-0.69]  < 0.001 | 0.62 [0.42-0.90]  0.013 | 0.81 [0.55-1.21]  0.306 | 0.52 [0.34-0.80]  0.003 |
| Everyday | 0.60 [0.42-0.84]  0.003 | 0.63 [0.45-0.88]  0.008 | 0.61 [0.43-0.87]  0.006 | 1.22 [0.86-1.73]  0.271 | 0.75 [0.52-1.08]  0.122 |
| Frequency of physical exercising during lockdowns (ref=Never) | *0.0003 | *0.0010 | *0.0531 | *0.0304 | *0.0001 |
| 1-2 days a week | 0.82 [0.68-0.98]  0.031 | 0.86 [0.71-1.03]  0.100 | 0.86 [0.72-1.04]  0.121 | 1.07 [0.89-1.29]  0.488 | 0.91 [0.74-1.12]  0.360 |
| 3-4 days a week | 0.71 [0.59-0.86]  < 0.001 | 0.77 [0.64-0.94]  0.008 | 0.83 [0.69-1.01]  0.059 | 0.83 [0.68-1.01]  0.062 | 0.67 [0.54-0.84]  < 0.001 |
| 5-6 days a week | 0.62 [0.50-0.77]  < 0.001 | 0.63 [0.51-0.79]  < 0.001 | 0.72 [0.58-1.25]  0.002 | 0.85 [0.68-1.07]  0.163 | 0.58 [0.45-0.75]  < 0.001 |
| Everyday | 0.66 [0.43-1.01]  0.055 | 0.65 [0.43-0.99]  0.044 | 0.85 [0.58-1.25]  0.420 | 0.73 [0.46-1.14]  0.160 | 0.59 [0.35-0.99]  0.046 |
| Frequency of recreational activities during lockdowns (ref=Never) | *0.0002 | *0.3029 | *0.0018 | *0.0618 |  |
| 1-2 days a week | 0.77 [0.65-0.91]  0.003 | 0.86 [0.72-1.02]  0.086 | 0.85 [0.72-1.01]  0.069 | 0.92 [0.78-1.10]  0.380 | - |
| 3-4 days a week | 0.74 [0.60-0.90]  0.003 | 0.82 [0.67-1.01]  0.065 | 0.73 [0.60-0.90]  0.003 | 0.75 [0.61-0.93]  0.008 | - |
| 5-6 days a week | 0.59 [0.44-0.78]  < 0.001 | 0.92 [0.69-1.21]  0.545 | 0.75 [0.57-0.98]  0.032 | 0.77 [0.57-1.03]  0.081 | - |
| Everyday | 0.59 [0.44-0.79]  < 0.001 | 0.80 [0.60-1.06]  0.120 | 0.61 [0.46-0.79]  < 0.001 | 0.82 [0.61-1.09]  0.173 | - |
| Keeping a routine during lockdowns (ref=Never) | *0.0001 | *0.0001 | *0.0001 | *0.0001 | *0.0001 |
| 1-2 days a week | 0.80 [0.63-1.01]  0.062 | 0.72 [0.56-0.91]  0.007 | 0.71 [0.55-0.91]  0.006 | 0.68 [0.53-0.86]  0.002 | 0.83 [0.63-1.08]  0.158 |
| 3-4 days a week | 0.57 [0.46-0.70]  < 0.001 | 0.70 [0.56-0.86]  0.001 | 0.68 [0.55-0.84]  < 0.001 | 0.59 [0.48-0.73]  < 0.001 | 0.67 [0.53-0.84]  0.001 |
| 5-6 days a week | 0.44 [0.36-0.55]  < 0.001 | 0.55 [0.44-0.68]  < 0.001 | 0.52 [0.42-0.65]  < 0.001 | 0.43 [0.34-0.53]  < 0.001 | 0.53 [0.41-0.68]  < 0.001 |
| Everyday | 0.46 [0.37-0.57]  < 0.001 | 0.56 [0.45-0.69]  < 0.001 | 0.53 [0.43-0.66]  < 0.001 | 0.43 [0.35-0.54]  < 0.001 | 0.58 [0.45-0.74]  < 0.001 |
| Frequency of meditation or praying during lockdowns (ref=Never) | *0.0057 | *0.0440 | *0.0116 | *0.0879 | *0.0217 |
| 1-2 days a week | 0.83 [0.71-0.98]  0.023 | 0.93 [0.79-1.09]  0.345 | 0.93 [0.80-1.08]  0.337 | 0.95 [0.81-1.12]  0.570 | 0.93 [0.77-1.13]  0.468 |
| 3-4 days a week | 1.05 [0.83-1.33]  0.685 | 1.30 [1.03-1.65]  0.029 | 1.37 [1.08-1.74]  0.008 | 1.01 [0.79-1.29]  0.954 | 1.28 [0.98-1.67]  0.073 |
| 5-6 days a week | 0.81 [0.57-1.15]  0.242 | 1.03 [0.74-1.45]  0.859 | 0.76 [0.55-1.05]  0.099 | 1.07 [0.75-1.51]  0.721 | 0.76 [0.49-1.18]  0.223 |
| Everyday | 0.65 [0.50-0.85]  0.002 | 0.82 [0.63-1.07]  0.143 | 0.99 [0.78-1.26]  0.940 | 0.68 [0.51-0.90]  0.006 | 0.69 [0.50-0.96]  0.026 |

Note: * = Walt test; n = number of participants; CI = Confidence Interval.

The cells with the “-” symbol represent that the independent variable was not associated with the outcome in the univariable analyses.

Each domain model was adjusted by sex and age.
